# Superior production of heavy pamamycin derivatives using a *bkdR* deletion mutant of *Streptomyces albus* J1074/R2

**DOI:** 10.1186/s12934-021-01602-6

**Published:** 2021-06-03

**Authors:** Lars Gläser, Martin Kuhl, Julian Stegmüller, Christian Rückert, Maksym Myronovskyi, Jörn Kalinowski, Andriy Luzhetskyy, Christoph Wittmann

**Affiliations:** 1 Institute of Systems Biotechnology, Saarland University, Saarbrücken, Germany; 2 Center for Biotechnology, Bielefeld University, Bielefeld, Germany; 3 Department of Pharmacy, Pharmaceutical Biotechnology, Saarland University, Saarbrücken, Germany

**Keywords:** Polyketide, Transcriptome, Metabolome, bkdR, l-valine, CoA thioester, Ethylmalonyl-CoA, Methylmalonyl CoA, Malonyl-CoA

## Abstract

**Background:**

Pamamycins are macrodiolides of polyketide origin which form a family of differently large homologues with molecular weights between 579 and 663. They offer promising biological activity against pathogenic fungi and gram-positive bacteria. Admittedly, production titers are very low, and pamamycins are typically formed as crude mixture of mainly smaller derivatives, leaving larger derivatives rather unexplored so far. Therefore, strategies that enable a more efficient production of pamamycins and provide increased fractions of the rare large derivatives are highly desired. Here we took a systems biology approach, integrating transcription profiling by RNA sequencing and intracellular metabolite analysis, to enhance pamamycin production in the heterologous host *S. albus* J1074/R2.

**Results:**

Supplemented with l-valine, the recombinant producer S. albus J1074/R2 achieved a threefold increased pamamycin titer of 3.5 mg L^−1^ and elevated fractions of larger derivatives: Pam 649 was strongly increased, and Pam 663 was newly formed. These beneficial effects were driven by increased availability of intracellular CoA thioesters, the building blocks for the polyketide, resulting from l-valine catabolism. Unfavorably, l-valine impaired growth of the strain, repressed genes of mannitol uptake and glycolysis, and suppressed pamamycin formation, despite the biosynthetic gene cluster was transcriptionally activated, restricting production to the post l-valine phase. A deletion mutant of the transcriptional regulator *bkdR,* controlling a branched-chain amino acid dehydrogenase complex, revealed decoupled pamamycin biosynthesis. The regulator mutant accumulated the polyketide independent of the nutrient status. Supplemented with l-valine, the novel strain enabled the biosynthesis of pamamycin mixtures with up to 55% of the heavy derivatives Pam 635, Pam 649, and Pam 663: almost 20-fold more than the wild type.

**Conclusions:**

Our findings open the door to provide rare heavy pamamycins at markedly increased efficiency and facilitate studies to assess their specific biological activities and explore this important polyketide further.

**Supplementary Information:**

The online version contains supplementary material available at 10.1186/s12934-021-01602-6.

## Background

**Fig. 1:**
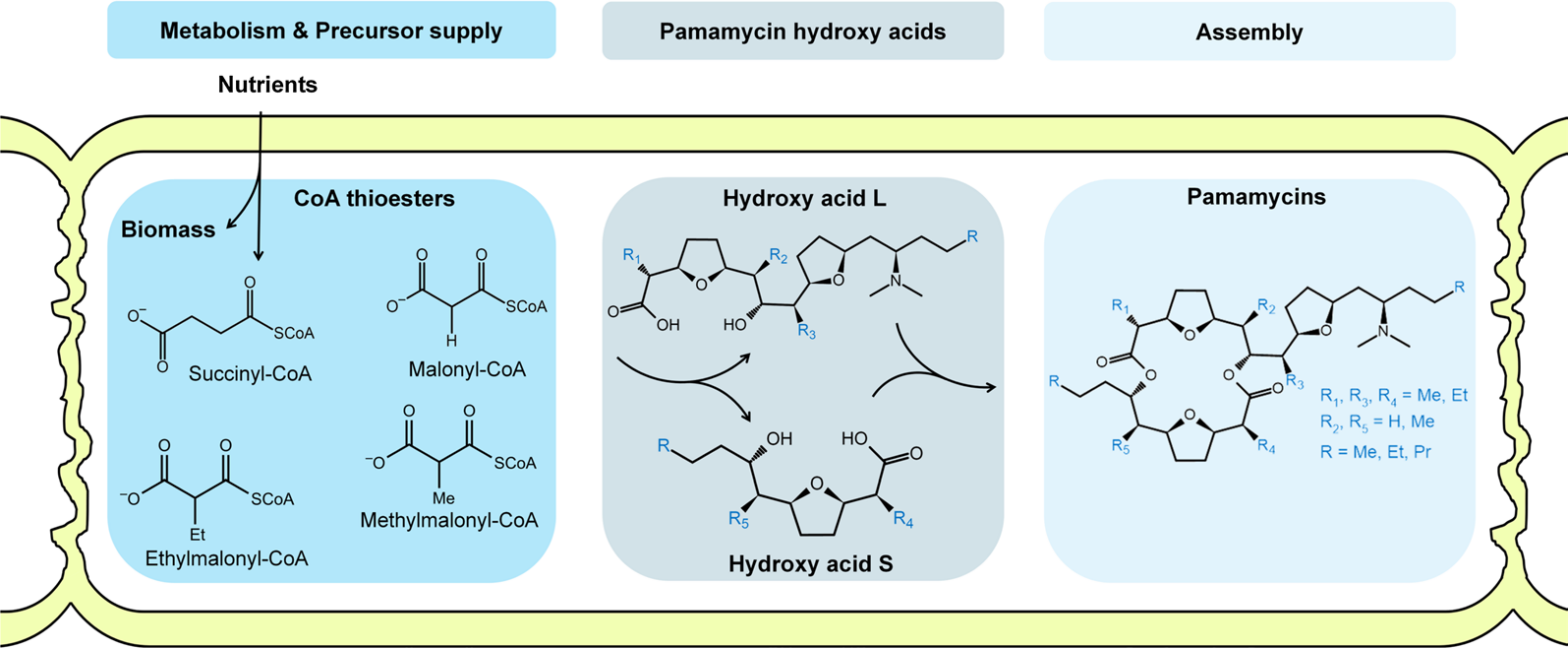
Biosynthesis of the polyketide pamamycin in *Streptomyces* [11]. Succinyl-CoA displays the starter unit for biosynthesis. Malonyl-CoA, methylmalonyl-CoA, and ethylmalonyl-CoA represent alternative extender units, which lead to the different derivatives of the polyketide. The incorporation of the CoA thioesters results in the two hydroxy acids L and S, which are finally assembled to yield pamamycin

Pamamycins, polyketide natural products with molecular weights between 579 and 663 (Fig. 1), were first isolated from the actinomycete *S. alboniger* ATCC 21461 [1], which accumulated Pam 621 as major component. Studies using *S. alboniger* IFO 12738 revealed the formation of a pamamycin mixture that mainly contained Pam 607 [2], later leading to the identification of 14 different homologues between 593 and 649 Da which differed in number and position of methyl and ethyl group substituents [3]. From an application viewpoint, pamamycins exhibit a range of interesting pharmacological activities. In actinomycetes, they induce the switch from substrate to aerial mycelium [2, 4, 5]. Moreover, they act against pathogenic fungi and gram-positive bacteria, including *Staphylococcus aureus* and multi-resistant clinical isolates of *Mycobacterium tuberculosis* [6], among the top ten causes of death worldwide with more than 1 million people that died from corresponding infections in 2019 [7]. These promising properties have raised the interest in pamamycins and stimulated a number of follow-up studies on physicochemical properties [3, 8, 9], mode-of-action [6, 10], and also synthetic routes over the past decades [9, 11]. Due to the high complexity and laboriousness, it was not feasible to use chemical synthesis for pamamycin production. On the other hand, fermentation methods were limited by low production levels and extraordinarily complex mixtures with up to 16 derivatives, mainly small ones [11–13]. Therefore, most of the biological activity studies were performed with the most abundant low-weight variants Pam 607 and Pam 621. Hereby, it was discovered that pamamycin derivatives differ in biological activity [3]. Because of inaccessibility, larger pamamycins have escaped further evaluation but remain highly interesting to be studied. In this regard, the development of strategies that (i) generally enable a more efficient formation of pamamycins and (ii) provide increased fractions of large derivatives for facilitated follow-up studies appears as promising direction of research.

Biochemically, pamamycin biosynthesis requires succinyl-CoA as the starter unit. The different pamamycin variants then result from promiscuous incorporation of malonyl-CoA, methylmalonyl-CoA, and ethylmalonyl-CoA as alternative extender units [11, 13], whereby the intracellular availability of the three CoA thioesters influences the formed spectrum [12, 13]. Interestingly, malonyl-CoA, methylmalonyl-CoA, and ethylmalonyl-CoA are provided from the degradation of branched-chain amino acids (BCAAs, i.e., l-valine, l-leucine, l-isoleucine). Following initial transamination of BCAAs into α-keto acids, decarboxylation, and dehydrogenation, catalyzed by the branched-chain amino acid dehydrogenase (BCDH) complex, yield the corresponding acyl-CoA derivatives [14]. Subsequently, these are converted into acetyl-CoA, propionyl-CoA, and succinyl-CoA, inter alia potentially leading to the pamamycin precursors malonyl-CoA, methylmalonyl-CoA, and ethylmalonyl-CoA, respectively [15–17]. The genome of *S. albus* contains the entire catabolic route for degradation of all three BCAAs [18] and the incorporation of l-valine-derived carbon into pamamycin has been experimentally shown [19].

Here, we modulated the branched-chain amino acid metabolism to improve the performance of the recombinant pamamycin producer *S. albus* J1074/R2. Supplementation of the medium with l-valine increased total pamamycin production and shifted the polyketide spectrum to heavier homologues, whereas l-isoleucine was found detrimental and l-leucine, like other amino acids did not result in a significant change. Systems-wide analysis of the l-valine-related effects, combining global transcription profiling and quantification of intracellular CoA thioesters revealed surprising dynamics: excess l-valine suppressed pamamycin biosynthesis but pre-conditioned *S. albus* towards a 3.5-fold increased production after l-valine depletion. On the transcriptional level, l-valine simultaneously perturbed primary and secondary metabolic pathways. This observation inspired the construction of a mutant that mimicked the l-valine effect on the genetic level. A *bkdR* deletion mutant, lacking a key regulator of the BCDH complex that presumably acted at the crossroad of primary and secondary metabolic pathways, provided a novel mode of decoupled pamamycin synthesis, apparently independent of the growth and nutrient status. The regulator mutant enabled the biosynthesis of pamamycin mixtures with up to 55% of the heavy-weight derivatives Pam 635, Pam 649, and Pam 663, almost 20-fold more than the wild type.

## Results

### l-Valine enhances pamamycin production in recombinant *S. albus* and shifts the spectrum to larger derivatives

**Fig. 2:**
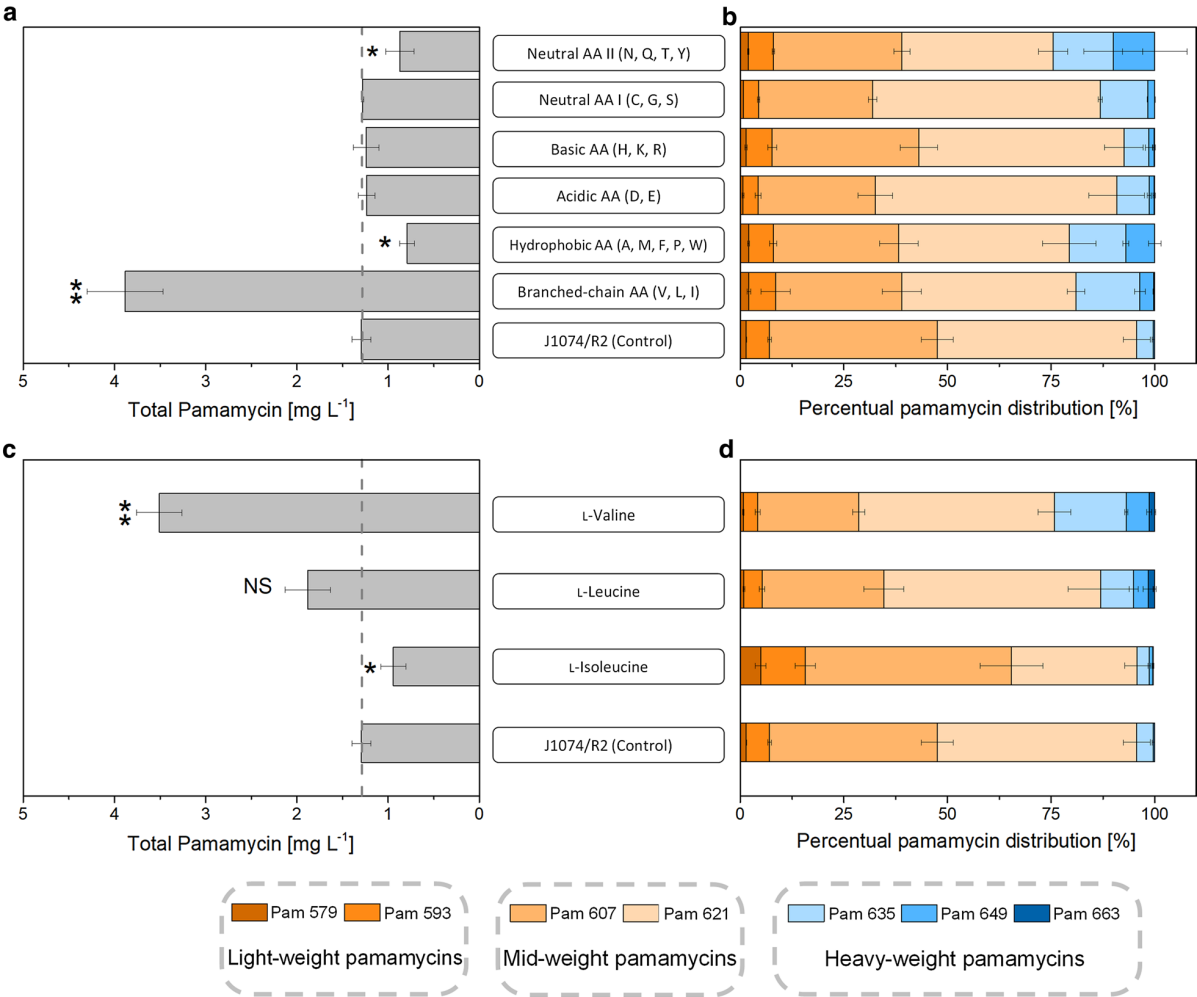
Impact of the nutrient environment on pamamycin production in *S. albus* J1074/R2. The strain was grown on a mineral mannitol-based medium containing 10 g L^−1^ (55 mM) of the sugar alcohol as sole carbon source (J1074/R2 Control), supplemented with different amino acid mixtures, containing three to five amino acids (designated by the one letter code) at a concentration of 3 mM each (**a**, **b**). The strain was grown on a mineral mannitol-based medium containing 10 g L^−1^ (55 mM) of the sugar alcohol as sole carbon source (J1074/R2 Control), supplemented individually with branched-chain amino acids (I, L, and V) at a concentration of 3 mM each (**c**, **d**). The data comprise the total pamamycin titer after 96 h (a, c), and the final pamamycin spectrum (**b**, **d**). Statistical significance was assessed by a t-test (*p* < 0.05, *; *p* < 0.01, **). n = 3

To assess its performance, the pamamycin producer *Streptomyces albus* J1074/R2 was cultivated in a minimal medium using 10 g L^−1^ mannitol as sole carbon source. The sugar alcohol is a frequently chosen carbon source to produce secondary metabolites [20–22]. The strain, harboring the pamamycin cluster from *S. alboniger* [11], accumulated pamamycin to a total titer of 1.3 mg L^−1^ after 48 h, whereby different derivatives between 579 and 649 Da were formed (Fig. 2a). The mid-weight pamamycins 607 and 621 were the dominant ones (Fig. 2b). They made up approximately 89%. The low weight pamamycins 579 and 593 contributed 7% to the total pool, whereas the fractions of the heavy-weight derivatives Pam 635 and Pam 649 were around 4%, respectively, and Pam 663, the largest derivative, was not observed (limit of detection < 1 µg L^−1^). A doubling of the mannitol content to 20 g L^−1^ did not affect production. The use of glucose as alternative carbon source resulted in a pamamycin titer of 0.9 mg L^−1^ (30% less than on mannitol), potentially due to negative effects of glucose-mediated carbon catabolite repression [23–25]. The addition of a casamino acid mixture (8 g L^−1^) increased the pamamycin titer after 48 h almost three-fold to 3.7 mg L^−1^. This observation indicated that nutrient specific effects rather than general carbon availability impacted polyketide production. Next, the influence of different amino acids was studied systematically. For this purpose, pamamycin production was compared for mixtures of three to five amino acids, each added at equimolar concentration (Fig. 2).

Among all experiments, only the BCAA mixture with l-valine, l-leucine, and l-isoleucine revealed a positive effect. The final pamamycin titer was increased to 4 mg L^−1^, whereby 20% of the product was composed of the heavy derivatives Pam 635, Pam 649, and Pam 663, whereby the latter variant was newly observed (Fig. 2a, b). The addition of acidic (D, E), basic (H, K, R), and neutral (C, G, S) amino acids did not affect the pamamycin level amount but slightly affected the product spectrum. A mixture of hydrophobic amino acids (A, M, F, P, W) and the second group of neutral amino acids (N, Q, T, Y) unfavorably decreased production to below 1 mg L^−1^. The branched-chain amino acids were now evaluated individually. Only the supplementation with l-valine revealed a positive effect: the total pamamycin level was increased to approximately 3.5 mg L^−1^ and the product spectrum was shifted towards heavier pamamycins of 635 to 663 Da (23%) (Fig. 2c, d). The addition of l-leucine did not significantly enhance production, whereas l-isoleucine was found even detrimental (Fig. 2c).

### The l-valine effect is complex and dynamic: l-valine suppresses pamamycin biosynthesis but primes *S. albus* to a production boost after its depletion

**Fig. 3:**
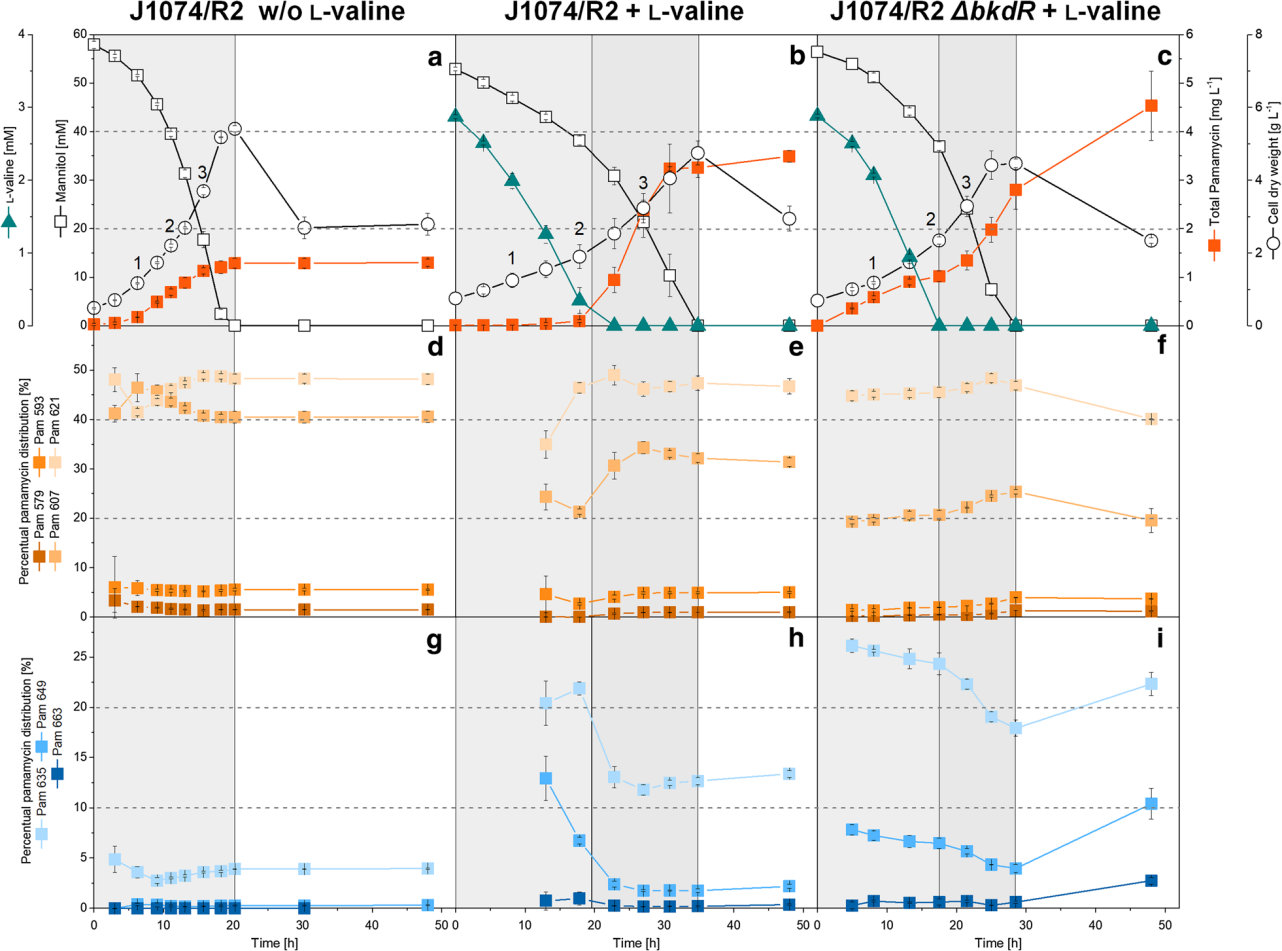
Influence of genetic and environmental perturbation on growth and pamamycin production in *Streptomyces albus* J1074/R2. The data show the time profiles for *Streptomyces albus* J1074/R2, grown on a mineral medium with 55 mM mannitol (**a**), and with 55 mM mannitol plus 3 mM l-valine (**b**), and the regulator null mutant *Streptomyces albus* J1074/R2 *ΔbkdR*, grown on a mineral medium with 55 mM mannitol plus 3 mM l-valine (c). The numbers given in the plots aside the growth data (1, 2, 3) indicate the three sampling time points for intracellular CoA ester analysis. n = 3

Given the ability of l-valine to stimulate pamamycin production, the effect of the amino acid on *S. albus* J1074/R2 was studied in more detail. First, we investigated the dynamics of production (Fig. 3). The control culture, containing only mannitol as carbon source, revealed a growth-coupled accumulation of pamamycin. The polyketide was formed from early on and reached a final titer of 1.3 mg L^−1^ after 21 h, when mannitol was depleted (Fig. 3a), and there was no further accumulation later. The pamamycin spectrum was constant over time and revealed Pam 607 (41%) and Pam 621 (48%) as dominant variants (Fig. 3d, g). During the process, *S. albus* J1074/R2 grew at a maximum specific growth rate of 0.13 h^−1^.

The addition of l-valine to the medium caused several effects (Fig. 3b). First, extracellular l-valine surprisingly suppressed pamamycin biosynthesis. Production of the polyketide was very weak if traces of the amino acid were still present, far below that of the control. Second, l-valine was co-consumed with mannitol, whereby it strongly suppressed the uptake of the sugar alcohol and reduced the maximum specific growth rate of the microbe by more than half to 0.05 h^−1^. This inhibition resulted in a pro-longed cultivation time of finally 35 h until all carbon was depleted, almost 70% more than in the control. Third, when l-valine had been completely consumed and mannitol remained as the sole carbon source, the cells switched to a highly productive mode and formed 3.5 (mg pamamycin) L^−1^ within only 16 h. The pamamycin space time yield during this phase (0.21 mg L^−1^ h^−1^) was 3.5-fold higher than that of the control (0.06 mg L^−1^ h^−1^), although the nutrient environment during this phase was apparently the same for both cultures. Fourth, regarding the pamamycin spectrum, the culture revealed two phases. The initial phase of weak production (12–18 h) formed high fractions of heavy pamamycins (Fig. 3h). Unfortunately, this had only a minor effect on the final spectrum due to the minute amounts formed during this period. After l-valine depletion, the product spectrum shifted to some extent from heavy to mid and light weight variants (Fig. 3e, h). Nevertheless, the relative (and absolute) production of the heavy pamamycins (Pam 635, Pam 649, Pam 663) was higher than in the control.

### l-Valine creates a memory effect on the metabolic level: CoA thioester availability is modulated even hours after the amino acid is depleted

**Fig. 4:**
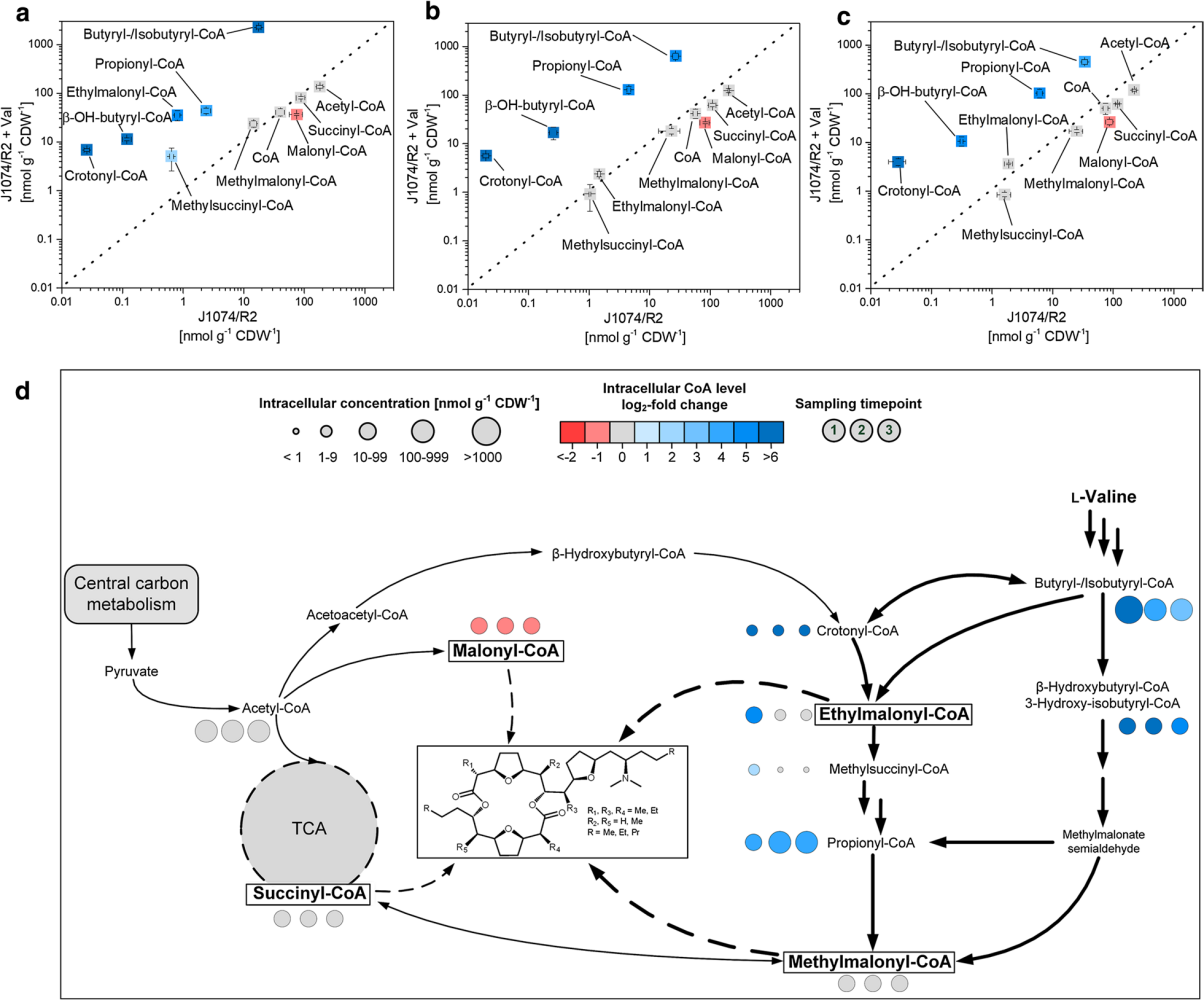
Dynamics of intracellular CoA thioesters during pamamycin production in *Streptomyces albus* J1074/R2. The data show the correlation of absolute levels between production on a mannitol-based medium (J1074/R2) and a mannitol-based medium, supplemented with 3 mM l-valine (J1074/R2 + Val) at three different timepoints (a, b, and c). In addition, the data from the l-valine supplemented process are mapped on the pathways of CoA thioester metabolism, whereby the size of the given circles represents the absolute concentrations at each timepoint (1, 2, 3), and the color represents the respective log_2_-fold change, as compared to the control J1074/R2 without the amino acid. n = 3

Pamamycin biosynthesis and l-valine degradation share CoA thioesters as pathway intermediates [12, 13]. To characterize the two routes and study their potential interaction, the CoA thioester spectrum in *S. albus* was quantified at three different time points during the l-valine supplemented process: growth under co-utilization of excess l-valine and mannitol (7 h, timepoint 1), weak pamamycin production in the presence of l-valine traces (18 h, timepoint 2), and strong production, 8 h after l-valine depletion (26 h, timepoint 3) (Fig. 3a, b, and Fig. 4a–c). A culture without l-valine addition was analyzed as control at the same time points.

Generally, l-valine supplementation strongly affected the CoA ester metabolism. During early growth (7 h), butyryl-/isobutyryl-CoA and hydroxybutyryl-/hydroxyisobutyryl-CoA, catabolic intermediates of l-valine degradation, were increased up to more than 100-fold as compared to the control and reached levels above 2000 nmol g^−1^ (Fig. 4a, d). In addition, several intermediates of the ethylmalonyl-CoA pathway, such as crotonyl-CoA, ethylmalonyl-CoA, methylsuccinyl-CoA, and propionyl-CoA were accumulated up to more than 30-fold, whereas malonyl-CoA was reduced by 50%. Interestingly, several of the increased CoA thioester pools remained high even hours after l-valine had been depleted. As an example, the level of butyryl-/isobutyryl-CoA remained above 500 nmol g^−1^ and displayed the dominant CoA thioester even 8 h after l-valine had been consumed (Fig. 4b, c). The pool of ethylmalonyl-CoA sharply dropped from 30 to below 5 nmol g^−1^ but was still slightly higher than in the control, whereas malonyl-CoA remained low. The observed changes substantially affected the ratio between the alternative extender units for pamamycin synthesis. In the l-valine supplemented process the ratio between malonyl-CoA, methylmalonyl-CoA, and ethylmalonyl-CoA was 100:65:97 during growth (7 h), 100:68:9 during weak production (18 h), and 100:64:14 in the major production phase (26 h), while it was 100:20:1, 100:28:2, and 100:29:2 at the corresponding time points in the control (Fig. 4).

### l-valine induces global transcriptional changes in *S. albus*

The recombinant producer was now studied on the transcriptional level. Using RNA sequencing, we analyzed the transcriptome of *S. albus* J1074/R2 in a l-valine supplemented process during initial growth (7 h) and strong pamamycin production (26 h) and sampled a process without l-valine addition as control. Sample-level quality control revealed excellent reproducibility (Additional file 1: Fig. S11, Additional file 1: Fig. S12). The individual replicates of all samples closely clustered together so that the observed expression differences could be fully attributed to the different experimental conditions. Various genes were significantly changed in expression by addition of l-valine during growth (7 h) (Additional file 1: Fig. S5). Interestingly, the gene expression pattern was still largely perturbed in the later process, although l-valine had been depleted approximately 8 h before and the resulting nutrient status (only mannitol present as carbon source), was the same for both processes at this time point.

**Fig. 5:**
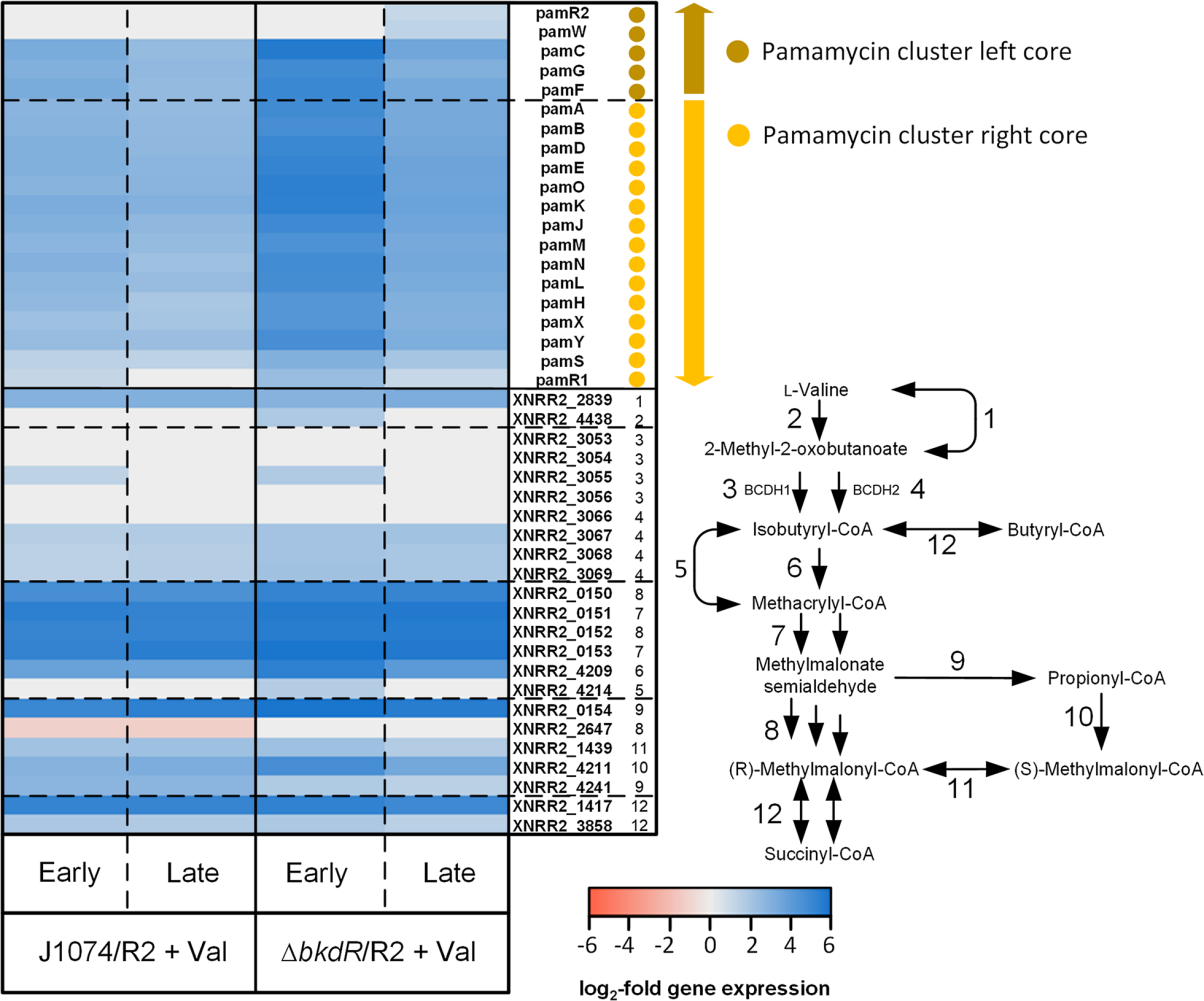
Influence of genetic and environmental perturbation on the expression of genes encoding the pamamycin biosynthetic cluster and l-valine degradation. The data represent the producer *Streptomyces albus* J1074/*R2* and the regulator mutant *Streptomyces albus* J1074/*R2 ΔbkdR*, grown on a mannitol-based medium, supplemented with 5 mM l-valine and sampled after 7 h (Early) and 18 h (Late). The pamamycin cluster genes are sorted, given their assignment to the left and right cluster core [13]. The l-valine degradation genes are sorted, based on their functional assignment to the pathway sequence, and are correspondingly labelled with colored circles. Gene expression of *Streptomyces albus* J1074/*R2* without l-valine (7 h) was set as a reference. n = 3

Surprisingly, l-valine triggered the overexpression of almost the entire pamamycin cluster (log_2_-fold change up to 3.2) (Fig. 5). This effect was observed for the growth phase (when pamamycin biosynthesis was strongly suppressed) and the production phase (when l-valine had long been depleted). As an exception, the pamamycin exporter (*pamW*) and its regulator *pamR2* were not affected. XNRR2_0579, XNRR2_1238, and XNRR2_5716, encoding phosphopantetheinyl transferases (PPTases) for initial activation of the acyl-carrier protein (*pamC*) during pamamycin synthesis, were found unchanged in expression (Additional file 1: Table S5). The catabolic l-valine route was strongly activated (Fig. 5), whereby BCDH II was identified as major complex catalyzing the initial step, and these changes remained over a period of 8 h after l-valine depletion. The genes XNRR2_0150 to XNRR2_0154, encoding for l-valine degradation from isobutyryl-CoA to methylmalonate semialdehyde, were among the 20 most upregulated genes (Table 1). Notably, excess l-valine did not inhibit expression of l-valine biosynthesis but rather upregulated certain steps of the anabolic route (Additional file 1: Table S3).

**Table 1:**
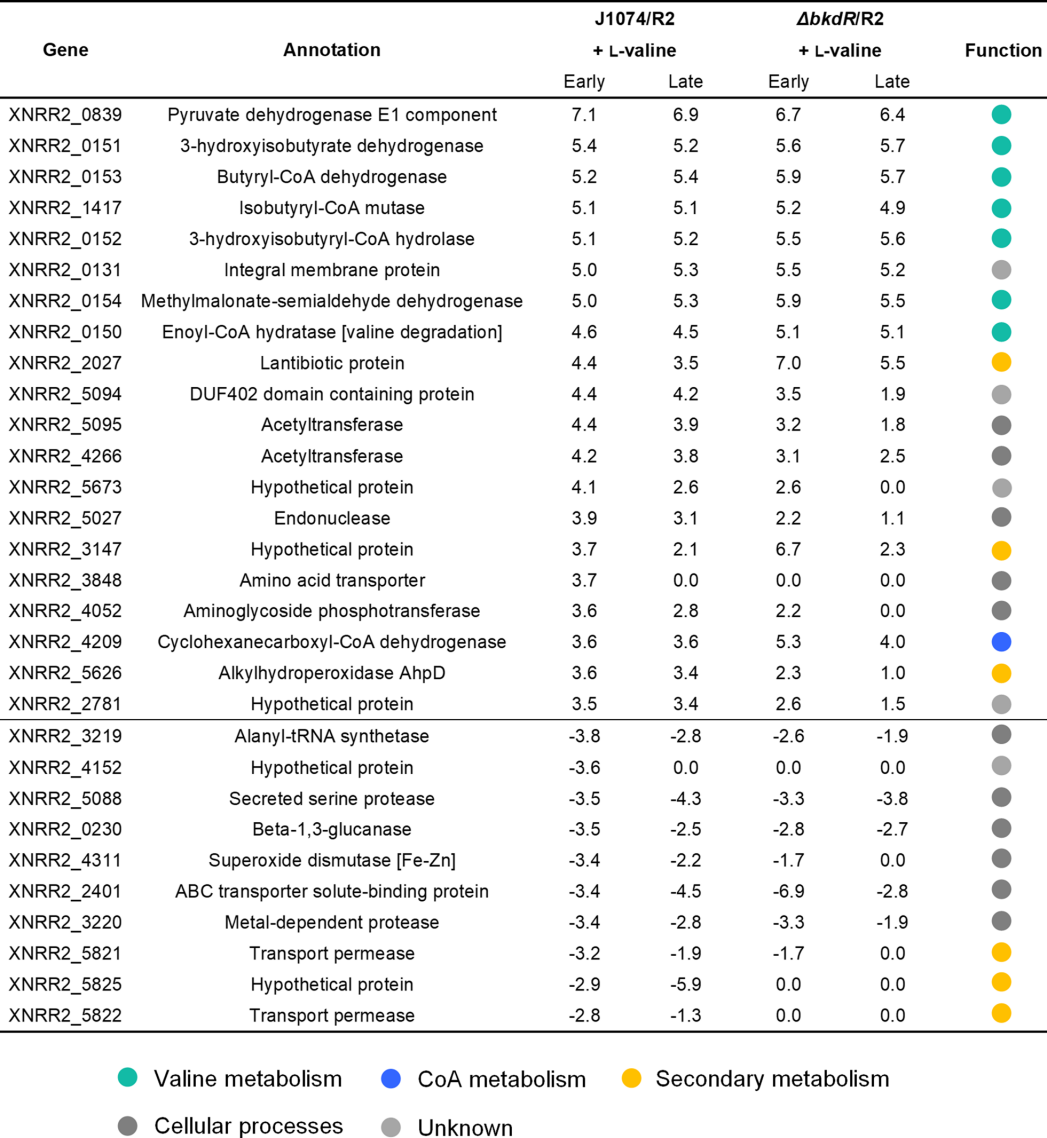
Most pronounced gene expression changes in *Streptomyces albus* J1074/R2 and *Streptomyces albus ΔbkdR*/R2 caused by supplementation of the minimal mannitol-based medium with l-valine

The data show the twenty most upregulated and the ten most downregulated genes Samples were taken from all cultures after 7 h (Early) and 18 h (Late). The expression level of the control culture J1074/R2 after 7 h was set as a reference. n = 3

Complex changes were observed for central carbon metabolism. As response to excess l-valine, cells exhibited a down-regulation of sugar transport genes encoding: the fructose-specific IIA/B/C component (XNRR2_0028) and the phosphor carrier protein HPr (XNRR2_0029) of the phosphotransferase system (PTS), and a sugar transporter with presumed function as mannitol permease (XNRR2_0970) (Additional file 1: Table S3). Additionally, two glycolytic genes, i. e. NADPH-dependent glyceraldehyde-3-phosphate dehydrogenase (XNRR2_0959) and pyruvate kinase (XNRR2_4449) were down-regulated (Additional file 1: Table S3). Slight expression changes resulted for reactions around the pools of acetyl-CoA and malonyl-CoA. As example, the fatty acid biosynthetic machinery, competing with pamamycin formation for malonyl-CoA was unchanged (Additional file 1: Table S3). However, other genes of CoA metabolism revealed a more pronounced expression change. Isobutyryl-CoA mutase was strongly activated (XNRR2_1417, log_2_-fold 5.2), and the initial steps of the ethylmalonyl-CoA pathway, involving acetyl-CoA carboxylase (XNRR2_1438, XNRR2_1987) and acetyl-/propionyl-CoA carboxylase (XNRR2_4211), were up-regulated too (Fig. 5). In contrast, crotonyl-CoA carboxylase/reductase (XNRR2_5889) revealed decreased expression (log_2_-fold -2.1). Regarding higher levels of cellular control, the addition of l-valine caused decreased gene expression of protein PII uridylyl transferase (XNRR2_1222 log_2_-fold-2.1), the nitrogen regulator protein P-II (XNRR2_1223, log_2_-fold change up to -2.1), an ammonium transporter (XNRR2_1224, log_2_-fold-2.3) and glutamine synthetase (XNRR2_4658, log_2_-fold-1.8), important genes of the nitrogen regulation system in *Streptomyces* [26, 27] (Additional file 1: Table S4). Furthermore, up to nine genes encoding sigma-factors and morphology regulators exhibited significantly changed expression (Additional file 1: Table S4). Finally, l-valine affected a range of pathways of secondary metabolism. In addition to the effects on pamamycin, the cluster encoding for the biosynthetic pathway paulomycin, a glycosylated antibiotic, was upregulated, whereas the clusters for the macrolide candicidin and the nine-membered bis-lactone antimycin were found decreased in expression (Additional file 1: Fig. S7).

### A null mutant of the *bkdR* regulator reveals decoupled pamamycin biosynthesis, apparently independent of the nutrient status

As shown, l-valine supplementation increased the final pamamycin titer. Despite this improvement, the set-up appeared suboptimal to some extent, because the amino acid caused poor growth of *S. albus* and the production of the polyketide was restricted to a short, post-l-valine phase so that the initial phase with high levels of methylmalonyl-CoA and ethylmalonyl-CoA as building blocks for the desired heavy pamamycins could not be exploited.

For optimization, we now aimed to break the (at least partly unfavorable) regulatory interactions. The transcriptome changes were too complex and provided too many targets to be systematically tackled within reasonable time, considering the demanding genetics of *S. albus*. Therefore, we were inspired to create a mutant that mimicked the promising l-valine effect on the genetic level and perturbed the crossroad between primary and secondary metabolic pathways. We decided to dissect cellular control at the level of *bkdR*, a transcriptional regulator of a branched-chain amino acid dehydrogenase complex which controls branched-chain amino acid metabolism, antibiotic production, and morphogenesis in *Streptomyces* [14, 28], presumably acting at the crossroad between the perturbed primary and secondary metabolic pathways. Therefore, we searched the genome of *S. albus* for a homolog of the known *bkdR* gene (SCO3832) from *S. coelicolor* [14]. The gene XNRR2_3053 showed 83% identity to SCO3832 (E-value 2E^−120^) and was assigned as the corresponding regulator *bkdR* in *S. albus* (Additional file 1: Figure S1). It turned out that *bkdR* was actively transcribed in the l-valine supplemented cultures and in the control cultures, whereby the expression level (10–20 sequencing reads in the different samples) was generally low. Subsequently, we deleted *bkdR* (XNRR2_3053) from the genome of *S. albus* J1074/R2. To this end, the linearized pKG1132hyg suicide vector was assembled in vitro with two 2000 bp fragments, containing the upstream and downstream flanking regions of the gene, respectively, using the primers 3053_HomA_Fw, 3053_HomA_Rev and 3053_HomB_Fw, and 3053_HomB_Rev (Additional file 1: Table S2). The plasmid was then transformed into *S. albus* using intergenic conjugation. The obtained *S. albus* exconjugants were evaluated by PCR using the primers 3053_ch_Fw and 3053_ch_Rev (Additional file 1: Table S2). Clones, which carried the desired deletion, revealed a shortened PCR fragment (739 bp), as compared to wildtype (1198 bp). One clone, additionally validated by sequencing for the desired deletion, was designated *S. albus ΔbkdR*/R2 and studied further (Additional file 1: Figure S2).

The *ΔbkdR* regulator mutant revealed substantially improved growth and production performance (Fig. 3). The formation of pamamycin occurred during all culture phases. It was no longer suppressed by l-valine but started immediately after inoculation, and it also was maintained during the stationary phase, when all carbon in the medium was exhausted. The final pamamycin level (4.5 mg L^−1^) was 1.5-fold higher than that of the wildtype with l-valine supplementation and almost fourfold higher than that of the wildtype without l-valine. The fraction of heavy pamamycins in the mixture (Pam 635, Pam 649, and Pam 663) was increased to 35%. During the stationary phase, the cells even formed a mixture with 55% of these large pamamycins (Additional file 1: Fig. S4). Notably, the *ΔbkdR* strain exhibited a 60% higher specific growth rate (*µ* = 0.08 h^−1^), than the wildtype.

### The deletion of *bkdR* beneficially activates the expression of genes related to pamamycin biosynthesis and sugar utilization

The *ΔbkdR* mutant revealed a drastic upregulation of the pamamycin cluster during the early growth phase (7 h, log_2_-fold up to 5.6) and the expression of the cluster remained much higher than in the parent producer during later stages, although it slightly dropped as compared to the start phase (Fig. 5). Notably, the repressing effects of l-valine on sugar uptake was diminished. The PTS genes (XNRR2_0028, XNRR2_0029), and the mannitol permease encoding gene (XNRR2_0970) were not downregulated in *ΔbkdR*, different to the wild type. In addition, other PTS components (XNRR2_5450, XNRR2_5451) were slightly upregulated (Additional file 1: Table S3). Genes associated to l-valine degradation showed no major difference in expression, matching the similar l-valine degradation rate in both strains (Fig. 5, Table 1). Related to morphogenesis, the mutant revealed modulated expression of several regulators, including XNRR2_1044 (sporulation transcription factor), XNRR2_3527 (BldN, RNA polymerase sigma-factor), XNRR2_2306 (Factor C protein), and different sigma factors (XNRR2_4476, XNRR2_5283) (Additional file 1: Table S4). Furthermore, the deletion of *bkdR* had significant effects on the expression of the clusters for paulomycin (no upregulation during later stages), candicidin and antimycin (no downregulation during later stages), and additionally it activated the expression of a lantibiotic cluster and a cluster, encoding for a so far unknown polyketide, during early growth (Additional file 1: Fig. S7, Cluster 12 and 26).

Regarding intracellular CoA thioesters, pools for acetyl-CoA, malonyl-CoA, propionyl-CoA, 3-hydroxy(iso-)butyryl-CoA, crotonyl-CoA (Additional file 1: Fig. S3), and ethylmalonyl-CoA were reduced during l-valine degradation, compared to J1074/R2, whereas the other esters remained unaffected. After l-valine had been depleted, acetyl-CoA and methylmalonyl-CoA exhibited slightly increased levels. The ratio between malonyl-CoA, methylmalonyl-CoA, and ethylmalonyl-CoA was 100:198:43 during l-valine degradation and 100:94:4 during later production.

## Discussion

### The feeding of l-valine enhances the production of rare heavy pamamycins: the formation of Pam 649 is increased up to sevenfold and Pam 663 appears as a newly formed derivative

Using *S. albus* J1074/R2 on a mannitol-based medium in this work (the defined medium was chosen to enable a clear monitoring of the medium supplementation effects), yielded a pamamycin mixture that well matched previous observations [1, 2, 11–13]: lower and mid weight pamamycins were dominating (95.7% Pam 579 to Pam 621), larger derivatives were contained only in low amount (4% Pam 635, 0.3% Pam 649), and the largest one Pam 663 was even absent (< 0.1%) (Figs. 2, 3). Remarkably, l-valine stimulated the formation of the larger pamamycins (Pam 635–Pam 663). The total production of Pam 649 was enhanced sevenfold (in J1074/R2) and 16-fold (in Δ*bkdR*/R2), as compared to the non-supplemented wild type. Considering the period of maximum formation (Δ*bkdR*/R2 during stationary phase), the fraction of this rare derivative was even increased 40-fold. Pam 663 was only formed, when l-valine was added. This shift of the product spectrum appears promising. It will help to overproduce rare heavy pamamycins, opening opportunities to study their so far uncharacterized specific biological activity to further explore this important polyketide [3, 6, 29].

### The deletion of *bkdR* improves the growth of *S. albus* J1074/R2 in the presence of l-valine and decouples pamamycin production from the nutrient status

As shown, the feeding of l-valine increased the final pamamycin titer but also revealed undesired side effects: impaired growth and suppression of pamamycin biosynthesis if the amino acid was present (Fig. 2, Fig. 3). It is interesting to note that such negative effects have been also observed in *S. ambofaciens* and *S. venezuelae*, where excess l-valine reduced cell growth and the production of spiramycin [30] and pikromycin [15]. Here, the reduced growth of *S. albus* obviously resulted from transcriptional repression of the mannitol PTS-mediated uptake and the glycolysis (Fig. 3b, Additional file 1: Table S3), similar to the l-valine related downregulation of glycolytic activity, observed in *B. subtilis* [15, 31]. Interestingly, the knockout of *bkdR* restored the PTS expression to high level and thereby eliminated the growth limitation, indicating at least an indirect connection between the regulator and the PTS. This link is rather unexplored in *Streptomyces*. Interestingly, previous studies of *Eubacterium limosum* and *Tepidanaerobacter acetatoxydans* suggest a possible regulatory function of *bkdR* on the PTS system: their Fis-family transcriptional regulators contain a HPr-like domain with significant homology to *bkdR* of *B. subtilis* [32]. Most importantly, the deletion of *bkdR* apparently impaired the control of pamamycin biosynthesis, enabling continuous polyketide formation, even after substrate depletion (Fig. 3, Fig. 5, Additional file 1: Fig. S4). Normally, secondary metabolism starts on the onset of aerial growth when nutrients become scarce [33] and branched-chain amino acids such as l-valine indicate a rich nutrient environment [34], and this control circuit is obviously destroyed by the *bkdR* deletion in *S. albus*. Simultaneously, the deletion of *bkdR* activated other secondary metabolite clusters in the presence of l-valine, (Additional file 1: Fig. S7), underlining the global role of this regulator, besides its well-known control of the BCDH cluster during amino acid degradation [14].

### The degradation of l-valine builds up a huge storage of intracellular CoA thioesters which lasts for a couple of hours after l-valine depletion and tunes the pamamycin spectrum

**Fig. 6:**
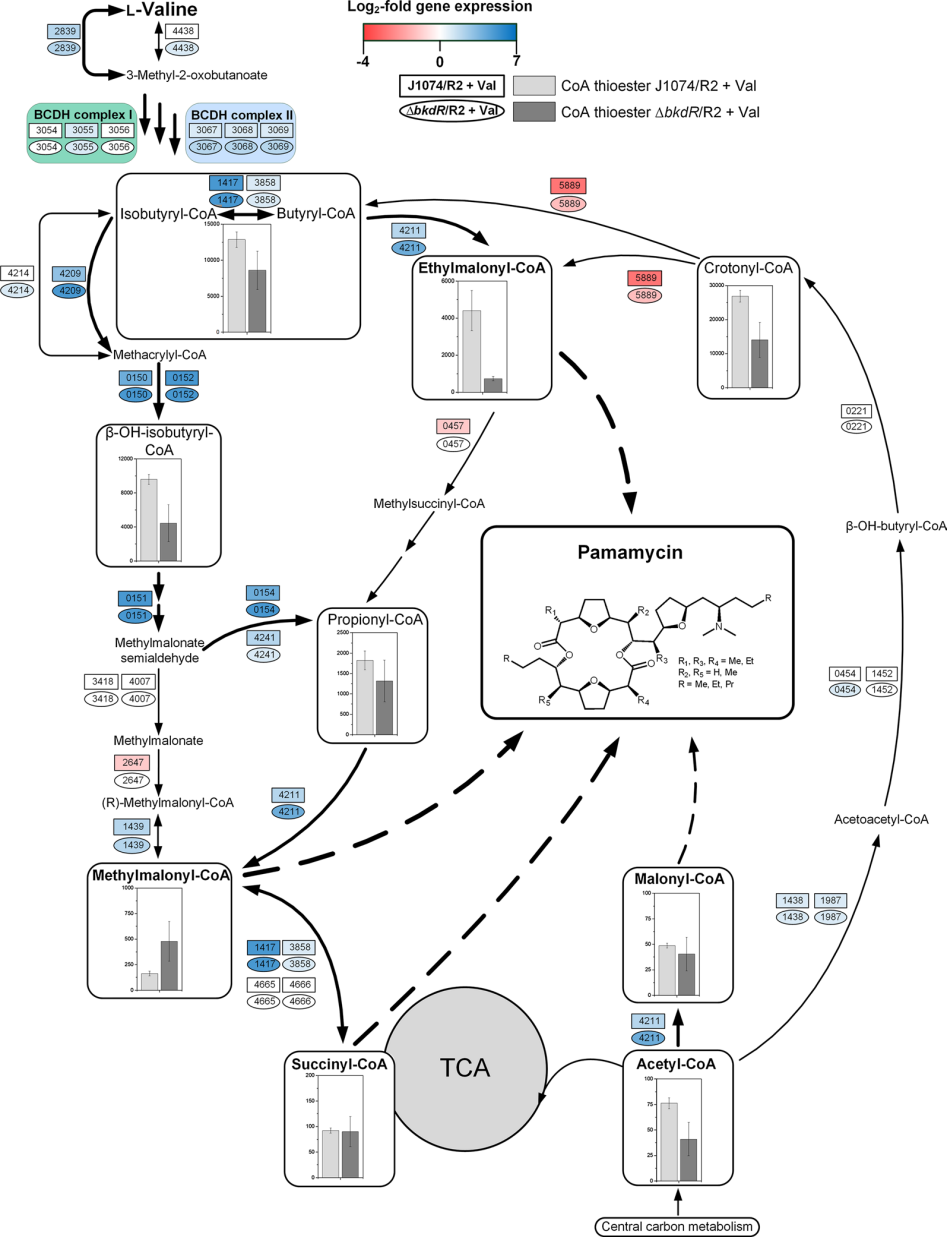
Multi-omics view on the effect l-valine on pathways of l-valine catabolism and CoA ester metabolism. The data comprise the differential gene expression of J10747/R2 (rectangle) and the mutant J1074/R2 *ΔbkdR* (ellipse) both supplemented with l-valine (7 h). The numbers indicate the corresponding genes (XNRR2_XXXX). The bar chart inserts display the relative intracellular CoA thioester levels, whereby J1074/R2, grown without l-valine (control), was set to 100%

l-Valine catabolism occurs via methylmalonate semialdehyde and methylmalonyl-CoA as central intermediates [13, 18] which well explains the elevated methylmalonyl-CoA pool, when l-valine was present (Fig. 6). The stimulating effect of l-valine on the pool of ethylmalonyl-CoA was not such obvious on a first glance. It seemed to involve the ethylmalonyl-CoA pathway, at least considering the increased abundance of the pathway intermediate crotonyl-CoA (Fig. 6) [13, 17]. In addition, we propose a second route for ethylmalonyl CoA supply based the transcriptome and metabolome data, which formed the CoA thioester from the catabolic l-valine intermediate isobutyryl-CoA via isomerization into butyryl-CoA and subsequent carboxylation into ethylmalonyl-CoA (Fig. 6). The two CoA thioester pools were strongly increased (Fig. 4, Fig. 6), and the expression of genes encoding for enzymes of this by-pass, isobutyryl-CoA mutase (XNRR2_1417, log_2_-fold 5.1) [35] and promiscuous acetyl-/propionyl-CoA carboxylases (e.g. XNRR2_4211/4212, log_2_-fold up to 4.7) was increased too (Fig. 6, Table 1). It was interesting to note that butyryl- and isobutyryl-CoA remained high, even hours after l-valine had been depleted and displayed a continuous reservoir to supply ethylmalonyl CoA. The increased abundance of methylsuccinyl-CoA under l-valine excess indicated significant loss of ethylmalonyl-CoA, caused by ethylmalonyl-CoA mutase (*meaA*). A deletion of this gene might enhance the ethylmalonyl-CoA pool even further, as previously shown for other polyketides in *S. venezuelae* [17]. In contrast, the exact reason of the reduced the malonyl-CoA pool under l-valine remains rather unclear. As recently discovered, the transcriptional regulator AccR controls several acetyl-CoA carboxylases and affects the levels of malonyl-CoA and methylmalonyl-CoA in *S. avermitilis* [36]. *S. albus* exhibits a homolog to this regulator: XNRR2_4213, annotated as TetR-family transcriptional regulator, exhibits a high similarity to AccR (E-value 2E^−160^) and the transcriptional regulator PccD from *Saccharopolyspora erythraea* which directly controls the BCDH operon [37]. Notably, the gene showed increased expression when l-valine was supplemented to the wildtype but remained unaffected in the *bkdR* mutant (Additional file Table S6). This observation can be taken as a first hint for a link between BCAA degradation, *bkdR* and the level of short-chain acyl-CoA esters but more work is needed in the future to fully resolve this picture in *S. albus.*

Clearly, the effects of l-valine were global and affected (i) morphology regulation and morphogenesis (Additional file 1: Table S4) [38], (ii) nitrogen assimilation and its control (Additional file 1: Table S4) [26, 27, 39, 40], and (iii) XNRR2_1071 (RelA) as part of the stringent response system [41, 42] (Additional file 1: Table S4). In addition, secondary metabolism was changed at the level of the clusters for paulomycin [43], candicidin [44], and antimycin [45] (Additional file 1: Fig. S7). Altogether, this indicates a complex regulatory network around l-valine [15, 46].

### The biosynthesis of pamamycin in *S. albus* involves l-valine mediated post-transcriptional control

Interestingly, l-valine activated the expression of the pamamycin biosynthetic gene cluster in *S. albus* J1074/R2, but simultaneously suppressed biosynthesis of the polyketide (Fig. 3, Fig. 5). These diametral effects indicate post-transcriptional control of pamamycin biosynthesis in the heterologous host. A possible mechanism could involve suppressed activation of the acyl-carrier protein *pamC*, the initial step of the pamamycin assembly, by phosphopantetheinyl transferase (PPtase) activity [47, 48]. The heterologous pamamycin cluster does not encode such an enzyme so that native PPtases apparently catalyzed the activation. The genome of *S. albus* contains three PPtase encoding genes, XNRR2_0579, XNRR2_1238, and XNRR2_5716. None of them was significantly affected in expression (Additional file 1: Table S5), so that we cannot provide a clear conclusion at this stage. However, *pamC* has been shown decisive for pamamycin synthesis [11]. The deletion of this gene *S. albus* J1074/R2 resulted in a dramatic reduction of pamamycin production, especially heavy ones. Remarkably, *pamC* was by far the strongest expressed gene of the pamamycin cluster in the *bkdR* deletion mutant (Fig. 6), eventually overriding the control so that the mutant accumulated pamamycin in the presence of l-valine (with a substantial fraction of large derivatives).

## Materials and methods

### Microorganisms and plasmids

The pamamycin producing strain *S. albus* J1074/R2 was obtained from previous work [11]. The amplification of transformation vectors during molecular cloning was conducted using *Escherichia coli* DH5α (Invitrogen, Carlsbad, USA). The methylation-sensitive strain *E. coli* ET12567 (*dam-13::Tn9*, *dcm-6*, *hsdM, hsdS*), containing the plasmid pUZ8002, was used as donor strain to conjugate DNA into *S. albus* [49]. Plasmid pKG1132, exhibiting β-glucuronidase reporter activity [50], was used to generate the integrative plasmid pKG1132hyg. All strains were maintained as glycerol stocks at − 80 °C.

### Molecular design and genetic engineering

First, DNA fragments from genomic DNA of *S. albus* J1074/R2 were amplified by PCR (2 × Phusion High-Fidelity PCR Master Mix with GC Buffer, Thermo Scientific, Waltham, MA, USA) using sequence specific primers (Additional file 1: Table S3). To clone the fragments into a linearized vector, homologous overlaps were created by fusing the forward and reverse primers with 20 nucleotide long sequences at their 5’ end. Subsequently, the fragments of interest were purified (Wizard SV Gel, PCR Clean-Up System, Promega, Mannheim, Germany) and assembled in vitro [51]. The vector backbone was linearized by EcoRV (FastDigest, Thermo Fisher Scientific, St. Leon-Roth, Germany) involving concomitant alkaline phosphatase treatment (Thermo Fischer Scientific). The reaction mixture for subsequent assembly of fragments and linearized vector contained 157.5 mM Tris·HCl (pH 7.5), 15.75 mM MgCl_2_, 15.75 mM DTT, 42 mg μL^−1^ PEG-800, 0.6 mg μL^−1^ NAD, 25 mU μL^−1^ Phusion High-Fidelity DNA Polymerase (Thermo Fisher Scientific), 7.5 mU μL^−1^ T5 exonuclease (Epicentre, Madison, USA), 4 U μL^−1^ Taq Ligase (Thermo Fisher Scientific), and 0.3 mM dNTPs. The obtained plasmid (Additional file 1: Table S2) was transferred into *E. coli* DH5α competent cells using heat shock, multiplied in the cloning host, isolated, and verified by restriction digestion. Subsequently, electrocompetent cells of *E. coli* ET12567/pUZ8002 were transformed with the correct plasmid and then used to transfer it into *S. albus* J1074/R2 by intergenic conjugation. For this purpose, the recipient strain was grown for four days for sporulation. Spores were washed off using sterile water, heat shocked for 10 min at 50 °C, mixed with *E. coli* ET12567/pUZ8002 (containing the recombinant plasmid) and plated on MS agar. After overnight incubation at 30 °C, plates were overlayed with phosphomycin (200 µg mL^−1^) and apramycin/hygromycin (50/100 µg mL^−1^). After four days of incubation at 30 °C, exconjugants were sprinkled with 3 µL X-Gluc (100 mg mL^−1^), incubated for 20–30 min at 30 °C, and evaluated for blue coloration. Blue stained exconjugants were passaged on MS agar plates containing phosphomycin. Spores were again washed off, diluted serially, and plated onto MS agar, supplemented with X-Gluc. White colonies, that had obviously undergone a second crossover, were evaluated by PCR to differentiate between the desired mutants and wild type. The software SnapGene (GSL Biotech LLC, San Diego, USA) was used for molecular strain, plasmid, and primer design.

### Media

For genetic engineering purposes, *E. coli* was grown in liquid Luria–Bertani medium (LB, Sigma-Aldrich, Darmstadt, Germany) or on solid LB medium containing 20 g L^−1^ agar (Becton Dickinson, Heidelberg, Germany), whereas *S. albus* J1074/R2 and its derivative strains were grown in liquid LB medium. To facilitate sporulation, *S. albus* was grown on mannitol-soy flour MS solid media containing per liter: 20 g mannitol (Sigma-Aldrich), 20 g soy flour (Schoenenberger Hensel, Magstadt, Germany) and 20 g agar (Becton Dickinson) [49]. For plasmid maintenance and selection, apramycin (50 µg mL^−1^), hygromycin (50 µg mL^−1^, *S. albus*, 100 µg mL^−1^, *E. coli*) and phosphomycin (200 µg mL^−1^) were added to the medium when needed. Additionally, for blue-white-screening, 5-Bromo-4-chloro-1H-indol-3-yl β-D-glucopyranosiduronic acid (X-Gluc) was supplemented to selection agar plates (40 µg mL^−1^) [52].

For pamamycin production, liquid pre-cultures of *S. albus* were grown in LB broth (20 g L^−1^) and main cultures were grown in basic minimal medium [12], which contained per liter: 10 g mannitol, 200 mM potassium phosphate buffer (pH 7.8), 15 g (NH_4_)_2_SO_4_, 1 g NaCl, 550 mg MgCl_2_*7H_2_O, 200 mg CaCl_2_, 30 mg 3,4-dihydroxybenzoic acid, 20 mg FeSO_4_, 2 mg FeCl_3_*6H_2_O, 2 mg MnSO_4_*H_2_O, 0.5 mg ZnSO_4_*H_2_O, 0.2 mg CuCl_2_*2H_2_O, 0.2 mg Na_2_B_4_O_7_*10H_2_O, 0.1 mg (NH_4_)_6_Mo_7_O_24_*4H_2_O, 1 mg nicotinamide, 1 mg riboflavin, 0.5 mg thiamine hydrochloride, 0.5 mg pyridoxine hydrochloride, 0.2 mg biotin, and 0.1 mg *p*-aminobenzoic acid. In addition, liquid media were amended with 30 g L^−1^ glass beads (soda-lime glass, 5 mm, Sigma-Aldrich) to avoid cell agglomeration. In selected experiments, single amino acids or mixtures of amino acids were added from filter sterilized stocks to the minimal medium, as stated above.

### Cultivation in shake flasks

Liquid cultures were incubated in baffled shake flasks (500 mL, 10% filling volume) on an orbital shaker (Multitron, Infors AG, Bottmingen, Switzerland, 5 cm shaking diameter, 230 rpm, 75% relative humidity), and at 28 °C. *S. albus* was incubated on MS agar at 28 °C for three days until sporulation occurred. Spores of a single colony were collected to inoculate the pre-culture, which was incubated overnight in LB medium. Afterwards, cells were collected (5,000 x*g*, 25 °C, 6 min), resuspended in main culture medium, and used to inoculate the main culture. All growth experiments were conducted as biological triplicate.

### Determination of cell concentration

The cell dry weight of the cultures was obtained by measuring the optical density at 600 nm, using the previously obtained correlation factor for *S. albus* of CDW (g L^−1^) = 0.62 × OD_600_ [12].

### Quantification of substrates

Mannitol was quantified by HPLC (1260 Infinity Series, Agilent, Darmstadt, Germany) using a Metacarb 87C column (300 × 7.8 mm, Agilent), a Metacarb 87C guard column (50 × 7.8 mm, Agilent), a desalting column (Microguard Deashing Cartridge, Bio-Rad, Munich, Germany), and demineralized water as mobile phase (85 °C, 0.6 mL min^−1^). Refraction index measurement was used for detection, and external standards were used for quantification [53, 54].

### Quantification of amino acids

The amino acids were quantified using HPLC with pre-column derivatization and fluorescence detection as described before [55]. For quantification, α-aminobutyric acid was used as internal standard [54].

### Extraction and quantification of pamamycins

Pamamycin analysis was performed following the protocol previously described [12]. In short, pamamycins were extracted with acetone and ethyl acetate, organic phase evaporated under nitrogen and the obtained extracts resolved in methanol. The filtered extracts were analyzed using LC–ESI–MS/MS (QTRAP 6500^+^, AB Sciex, Darmstadt, Germany) coupled to an HPLC system (Agilent Infinity 1290 System). Analytes were separated on a C18 column (Vision HT C18 HighLoad, 100 mm × 2 mm, 1.5 µm, Dr. Maisch, Ammerbuch-Entringen, Germany) at 45 °C and a flow rate of 300 µL min^−1^ (8 mM ammonium formate in 92% acetonitrile). Detection was carried out in positive selected ion monitoring (SIM) mode, using the [M + H]^+^ ion for each pamamycin derivative.

### Extraction of intracellular CoA thioesters

CoA thioesters were extracted using the previously established protocol [12]. In short, a broth sample (approximately 8 mg CDW) was collected and immediately transferred into a pre-cooled extraction and quenching buffer (95% acetonitrile, 25 mM formic acid, − 20 °C). Simultaneously a fully ^13^C-enriched CoA thioester standard was added during harvesting for later absolute quantification. The volume ratio was 1:4. The obtained solution was thoroughly mixed while cooled on ice for 10 min, and then clarified from debris (15,000×*g*, 4 °C, 10 min). The obtained supernatant was mixed with 10 mL super cooled deionized water (− 2 °C). The cell pellet was twice washed with 8 mL super cooled deionized water. Afterwards, all supernatants were combined, frozen with liquid nitrogen, freeze-dried, and then re-dissolved in 500 µL pre-cooled resuspension buffer (25 mM ammonium formate, pH 3.0, 2% MeOH, 4 °C). The buffered extract was filtered (Ultrafree-MC 0.22 µm, Merck, Millipore, Germany) prior to analysis.

### Quantification of CoA thioesters using LC–ESI–MS/MS

Analysis of the CoA thioesters were performed as described before [12]. Therefore, the extracts were analyzed on a core–shell reversed phase column (Kinetex XB-C18, 100 × 2.1 mm, 2.6 µ, 100 Å, Phenomenex) was applied at 40 °C, using a gradient of formic acid (50 mM, adjusted to pH 8.1 with ammonium hydroxide 25% in H_2_O, eluent A) and methanol (eluent B) at a flow rate of 300 µL min^−1^. The fraction of eluent B was as follows: 0–7 min, 0–10% B; 7–10 min, 10–100% B; 10–11 min, 100% B; 11–12 min, 100–0% B; 12–15 min, 0% B. During the first 3 min of the analysis, the outflow from the chromatographic column was discharged to minimize the entry of salts from samples into the mass spectrometer. The individual CoA thioesters were detected using multiple reaction monitoring (MRM), involving the corresponding parent ion and its respective daughter ion.

### Transcriptomic analysis

Cells were collected by centrifugation (20,000×*g*, 4 °C, 1 min), and the obtained pellet was immediately frozen in liquid nitrogen. Total RNA was isolated from 3 biological replicates per strain using a Quick-RNA Miniprep Plus kit according to the manufacturer’s instructions (Zymo Research). After additional DNase treatment, RNA samples were purified with an RNA Clean&Concentrator-5 kit (Zymo Research) and quantified with a DropSense 16 (Trinean NV). The quality of total RNA was controlled with an RNA 6000 Nano kit in an Agilent 2100 Bioanalyzer (Agilent Technologies). To construct whole transcriptome cDNA libraries, 2.5 μg total RNA each (RIN > 9) was used for the depletion of rRNA with a Ribo-Zero rRNA Removal Kit (Bacteria) according to manufacturer’s instructions (Illumina). The rRNA removal was checked with an Agilent RNA Pico 6000 kit and the Agilent 2100 Bioanalyzer (Agilent Technologies). The mRNA obtained was converted to a cDNA library according to the TruSeq Stranded mRNA Sample Preparation guide (Illumina). The quality and quantity of the cDNA library was checked with an Agilent High Sensitivity DNA kit and the Agilent 2100 Bioanalyzer (Agilent Technologies). Sequencing was performed on an Illumina NextSeq 500 using 75 bases read length (Illumina).

Reads were mapped to the *S. albus* J1074/R2 genome sequence (CP059254.1) with Bowtie2 using standard settings [56] except for increasing the maximal allowed distance for paired reads to 600 bases. For visualization of read alignments, ReadXplorer 2.2.3 [57] was used. For counting of reads mapping to gene features, FeatureCounts v.2.0.0 [58] was applied using the parameters -M -O and -s 1. Using the resulting data, DESeq2 [59] was used to QC the datasets via, among others, calculation of the sample-to-sample distances (Additional file 1: Fig. S11) and PCA (Additional file 1: Fig. S12). In addition, DESeq2 was used to calculate DGE datasets. Raw datasets (sequenced reads) as well as processed datasets (input matrix and normalized read counts from DESeq2) are available from GEO (GSE168592). For statistical analysis, Student's t test was carried out and the data were filtered for genes with a log2‐fold change ≥ 1 (p ≤ 0.05). Data analysis and visualization was conducted using the software package gplots [60, 61].

## Supplementary Information

**Additional file 1:** Additional figures S1 to S12 and tables S1 to S6.

## Data Availability

The dataset(s) supporting the conclusions of this article are all included within the article.
