# Supplementary material for: Superior production of heavy pamamycin derivatives using a bkdR deletion mutant of Streptomyces albus J1074/R2
Source: Microb Cell Fact. 2021 Jun 3;20:111. doi: 10.1186/s12934-021-01602-6 (PMC8176718; doi:10.1186/s12934-021-01602-6)
Supplement: Supplementary file 1 — Additional file 1: Additional figures S1 to S12 and tables S1 to S6. [file 12934_2021_1602_MOESM1_ESM.docx]

Additional File for

**Superior production of heavy pamamycin derivatives using a *bkdR* null mutant of *Streptomyces albus***

Submitted for publication in *Microbial Cell Factories*

Lars Gläser^1^, Martin Kuhl^1^, Julian Stegmüller^1^, Christian Rückert^2^, Jörn Kalinowski^2^, Andriy Luzhetskyy^3^, and Christoph Wittmann^1#^

^1^Institute of Systems Biotechnology, Saarland University, Saarbrücken, Germany

^2^Center for Biotechnology, Bielefeld University, Bielefeld, Germany

^3^Department of Pharmacy, Pharmaceutical Biotechnology, Saarland University, Saarbrücken, Germany

Contact information

[lars.glaeser@uni-saarland.de](mailto:lars.glaeser@uni-saarland.de)

[martin.kuhl@uni-saarland.de](mailto:martin.kuhl@uni-saarland.de)

[julian.stegmüller@uni-saarland.de](mailto:julian.stegmüller@uni-saarland.de)

[cruecker@cebitec.uni-bielefeld.de](mailto:cruecker@cebitec.uni-bielefeld.de)

jkalinowski@cebitec.uni-bielefeld.de

[a.luzhetskyy@mx.uni-saarland.de](mailto:a.luzhetskyy@mx.uni-saarland.de)

[christoph.wittmann@uni-saarland.de](mailto:christoph.wittmann@uni-saarland.de)

^#^Corresponding author: Phone: +49 681 302 71970, FAX: +49 681 302 71972, e-mail: [christoph.wittmann@uni-saarland.de](mailto:christoph.wittmann@uni-saarland.de)

Table S1: Strain and plasmids used for performing deletion mutants.

| **Strains** | **Description** | **Reference** |
| --- | --- | --- |
| E. coli ET12567 (pUZ8002) | Cells for conjugative DNA transfer | [[1](#_ENREF_1)] |
| **Plasmids** | **Description** | **Reference** |
| pKG1132 | Suicide vector for genome-based modifications. comprising a MCS. an ORI for *E. coli*. and *AmR* and *gusA* as selection marker | [[2](#_ENREF_2)] |
| pKG1132hyg | Suicide vector for genome-based modifications. comprising a MCS. an ORI for *E. coli*. and *HygR* and *gusA* as selection marker | This work |
| pKG1132hyg_*ΔbdkR* | Suicide vector for the deletion of the gene XNRR2_3053 | This work |

Table S2: Primers for genome-based engineering of *S. albus* J1074/R2

| **Name** | **Sequence** |
| --- | --- |
| 3053_HomA Fw | 5’-GATCCGCGGCCGCGCGCGATtcctgccgacggtcttcctg-3‘ |
| 3053_HomA Rev | 5’-agtggggagcgatacgaatgtgacgcgaaccgggccgcag-3‘ |
| 3053_HomB Fw | 5’-ctgcggcccggttcgcgtcacattcgtatcgctccccactc-3‘ |
| 3053_HomB Rev | 5’-GACATGATTACGAATTCGATcttcgaccagtacagccgcttg-3‘ |
| 3053_ch_Fw | 5’-ggatgatcaactcggtggtcg-3‘ |
| 3053_ch_Rev | 5’-cggaacggggactcgtcg-3‘ |
| Seq1_DbdkR_Fw | 5’-gctcgatccgtggcatcc-3‘ |
| Seq2_DbdkR_Fw | 5’-aggtctccagctacaacctg-3‘ |
| Seq3_DbdkR_Fw | 5’-cgaacaatcggtaggtgagg-3‘ |

Table S3: Gene expression of identified genes participating in biosynthesis of fatty acids, cofactors biotin, pantothenate, and CoA, branched-chain amino acids, as well as participating in phosphotransferase system (PTS) and at the entrance to central carbon metabolism in heterologous pamamycin producing *Streptomyces albus* J1074/R2 and *Streptomyces albus* *ΔbkdR*/R2 with supplemented l-valine in mannitol minimal medium during early growth (Early) and main production phase (Late). The expression level of the wildtype culture (J1074/R2) in minimal medium without amino acids during early growth (7h) was set as a reference (Control). n = 3

| **Gene** | **Annotation** | **Control** | | **J1074/R2**  **+ Valine** | | ***ΔbkdR*/R2**  **+ Valine** | | |
| --- | --- | --- | --- | --- | --- | --- | --- | --- |
|  |  | **Late** | **Early** | | **Late** | | **Early** | **Late** |
| **Fatty acid biosynthesis (FAS)** | | | | | | | | |
| XNRR2_0316 | acyl-ACP desaturase, Stearoyl-ACP desaturase | 0.0 | 0.0 | | 1.1 | | 0.0 | 0.0 |
| XNRR2_0345 | Long-chain-fatty-acid--CoA ligase | 0.0 | 0.0 | | 0.0 | | 0.0 | 0.0 |
| XNRR2_0396 | 3-oxoacyl-[acyl-carrier-protein] synthase, KASIII | 0.0 | 0.0 | | 0.0 | | 0.0 | 0.0 |
| XNRR2_0403 | Long-chain-fatty-acid--CoA ligase | 0.0 | -1.5 | | -1.4 | | 0.0 | 0.0 |
| XNRR2_0583 | acyl-CoA synthase | 0.0 | 0.0 | | 0.0 | | 1.4 | 0.0 |
| XNRR2_0588 | 3-oxoacyl-ACP synthase III | 0.0 | 0.0 | | 1.0 | | 0.0 | 0.0 |
| XNRR2_0913 | short chain dehydrogenase/reductase family oxidoreductase | 0.0 | 0.0 | | 0.0 | | 0.0 | 0.0 |
| XNRR2_3581 | 3-oxoacyl-[acyl-carrier protein] reductase | 0.0 | 0.0 | | 0.0 | | 2.7 | 0.0 |
| XNRR2_3583 | Acyl-CoA synthetase | 0.0 | 0.0 | | 0.0 | | 0.0 | 0.0 |
| XNRR2_4019 | Acyl-CoA carboxylase complex A subunit | 0.0 | 0.0 | | 0.0 | | 0.0 | 0.0 |
| XNRR2_4211 | Acetyl/propionyl CoA carboxylase alpha subunit | 0.0 | 2.8 | | 3.1 | | 4.6 | 3.4 |
| XNRR2_4377 | Acyl-CoA synthetase | 0.0 | 0.0 | | 0.0 | | 1.1 | 0.0 |
| XNRR2_4461 | Short-chain dehydrogenase/reductase SDR | 0.0 | 0.0 | | 0.0 | | 2.5 | 0.0 |
| XNRR2_4509 | 3-oxoacyl-[acyl-carrier-protein] synthase 2 | 0.0 | 0.0 | | 0.0 | | 0.0 | 0.0 |
| XNRR2_4511 | 3-oxoacyl-[acyl-carrier-protein] synthase 3 | 0.0 | 0.0 | | 0.0 | | 0.0 | 0.0 |
| XNRR2_4512 | FabD | 0.0 | 0.0 | | 0.0 | | 0.0 | 0.0 |
| XNRR2_4749 | Acyl-CoA synthetase | 0.0 | 0.0 | | 0.0 | | 0.0 | 0.0 |
| XNRR2_4989 | Oxidoreductase | 0.0 | 0.0 | | 0.0 | | 0.0 | 0.0 |
| XNRR2_5007 | 3-oxoacyl-[acyl-carrier-protein] reductase | 0.0 | 0.0 | | 0.0 | | 0.0 | 0.0 |
| XNRR2_5131 | Acyl-CoA synthetase | 0.0 | 0.0 | | 0.0 | | 0.0 | 0.0 |
| XNRR2_5492 | 3-oxoacyl-[acyl-carrier protein] reductase | 0.0 | 0.0 | | 0.0 | | 0.0 | 0.0 |
| XNRR2_5537 | 3-oxoacyl-[acyl-carrier protein] reductase | 0.0 | 0.0 | | 0.0 | | 0.0 | 0.0 |
| **Biotin biosynthesis** | | | | | | | | |
| XNRR2_4025 | Biotin-protein ligase | 0.0 | 0.0 | | 0.0 | | 0.0 | 0.0 |
| XNRR2_5558 | Dethiobiotin synthetase | 0.0 | 0.0 | | 0.0 | | 0.0 | 0.0 |
| XNRR2_5559 | Adenosylmethionine-8-amino-7-oxononanoate aminotransferase | 0.0 | 0.0 | | 0.0 | | 0.0 | 0.0 |
| XNRR2_5560 | Biotin synthase | 0.0 | 0.0 | | 0.0 | | 0.0 | 0.0 |
| XNRR2_5561 | 8-amino-7-oxononanoate synthase | 0.0 | 0.0 | | 0.0 | | 0.0 | 0.0 |
| XNRR2_4025 | Biotin-protein ligase | 0.0 | 0.0 | | 0.0 | | 0.0 | 0.0 |
| XNRR2_5558 | Dethiobiotin synthetase | 0.0 | 0.0 | | 0.0 | | 0.0 | 0.0 |
| **Branched-chain amino acid biosynthesis** | | | | | | | | |
| XNRR2_0081 | Threonine dehydratase | 0.0 | 0.0 | | 0.0 | | 0.0 | 0.0 |
| XNRR2_0356 | Thiamine pyrophosphate-requiring enzyme | 0.0 | 0.0 | | 0.0 | | 0.0 | 0.0 |
| XNRR2_1254 | 3-isopropylmalate dehydratase small subunit | 0.0 | 0.0 | | 0.0 | | 0.0 | 0.0 |
| XNRR2_1255 | 3-isopropylmalate dehydratase large subunit | 0.0 | 0.0 | | 0.0 | | 0.0 | 0.0 |
| XNRR2_1299 | (R)-citramalate synthase | 0.0 | 3.3 | | 2.9 | | 2.5 | 2.5 |
| XNRR2_1304 | Branched-chain amino acid aminotransferase | 0.0 | 0.0 | | 0.0 | | 0.0 | 0.0 |
| XNRR2_1305 | 3-isopropylmalate dehydrogenase | 0.0 | 0.0 | | 0.0 | | 0.0 | 0.0 |
| XNRR2_1319 | Ketol-acid reductoisomerase | 0.0 | 1.5 | | 1.6 | | 1.7 | 1.5 |
| XNRR2_1320 | Acetolactate synthase small subunit | 0.0 | 2.1 | | 2.1 | | 2.0 | 2.0 |
| XNRR2_1321 | Acetolactate synthase large subunit | 0.0 | 2.4 | | 2.2 | | 2.2 | 2.2 |
| XNRR2_2513 | Aminotransferase AlaT | 0.0 | 0.0 | | 0.0 | | 0.0 | 0.0 |
| XNRR2_3281 | Threonine dehydratase | 0.0 | 0.0 | | 0.0 | | 0.0 | 0.0 |
| XNRR2_3504 | Dihydroxy-acid dehydratase | 0.0 | 0.0 | | 0.0 | | 0.0 | 0.0 |
| XNRR2_4041 | Threonine dehydratase | 0.0 | 0.0 | | 0.0 | | 0.0 | 0.0 |
| XNRR2_4064 | Aspartate aminotransferase | 0.0 | 1.1 | | 0.0 | | 0.0 | 0.0 |
| XNRR2_4225 | Acetolactate synthase | 0.0 | 0.0 | | 0.0 | | 0.0 | 0.0 |
| XNRR2_4410 | 2-isopropylmalate synthase | 0.0 | 0.0 | | 0.0 | | 0.0 | 0.0 |
| XNRR2_4438 | Branched-chain amino acid aminotransferase | 0.0 | 0.0 | | 0.0 | | 1.8 | 0.0 |
| XNRR2_5309 | Aminodeoxychorismate lyase | 0.0 | 0.0 | | 0.0 | | 0.0 | 0.0 |
| **Pantothenate and CoA biosynthesis** | | | | | | | | |
| XNRR2_0356 | Thiamine pyrophosphate-requiring enzyme | 0.0 | 0.0 | | 0.0 | | 0.0 | 0.0 |
| XNRR2_0487 | Dihydropyrimidinase | 0.0 | 1.2 | | 0.0 | | 2.1 | 0.0 |
| XNRR2_0488 | N-carbamoylputrescine amidase / Omegaamidase (Nit2-like protein) | 0.0 | 0.0 | | 0.0 | | 1.9 | 0.0 |
| XNRR2_0579 | 4'-phosphopantetheinyl transferase | 0.0 | 0.0 | | 0.0 | | 0.0 | 0.0 |
| XNRR2_1238 | Phosphopantetheine adenylyltransferase | 0.0 | 0.0 | | 0.0 | | 0.0 | 0.0 |
| XNRR2_1304 | Branched-chain amino acid aminotransferase | 0.0 | 0.0 | | 0.0 | | 0.0 | 0.0 |
| XNRR2_1319 | Ketol-acid reductoisomerase | 0.0 | 1.5 | | 1.6 | | 1.7 | 1.5 |
| XNRR2_1320 | Acetolactate synthase small subunit | 0.0 | 2.1 | | 2.1 | | 2.0 | 2.0 |
| XNRR2_1321 | Acetolactate synthase large subunit | 0.0 | 2.4 | | 2.2 | | 2.2 | 2.2 |
| XNRR2_3418 | Aldehyde dehydrogenase | 0.0 | 0.0 | | 0.0 | | 0.0 | 0.0 |
| XNRR2_3473 | Pantoate--beta-alanine ligase | 0.0 | 0.0 | | 0.0 | | -1.5 | 0.0 |
| XNRR2_3476 | Type III pantothenate kinase | 0.0 | 0.0 | | 0.0 | | 0.0 | 0.0 |
| XNRR2_3504 | Dihydroxy-acid dehydratase | 0.0 | 0.0 | | 0.0 | | 0.0 | 0.0 |
| XNRR2_3726 | Aspartate 1-decarboxylase | 0.0 | 0.0 | | 0.0 | | 0.0 | 0.0 |
| XNRR2_3763 | Pantothenate kinase | 0.0 | 1.0 | | 0.0 | | 0.0 | 0.0 |
| XNRR2_3772 | Holo-[acyl-carrier protein] synthase | 0.0 | 0.0 | | 0.0 | | 0.0 | 0.0 |
| XNRR2_4007 | Aldehyde dehydrogenase | 0.0 | 0.0 | | 0.0 | | 0.0 | 0.0 |
| XNRR2_4225 | Acetolactate synthase | 0.0 | 0.0 | | 0.0 | | 0.0 | 0.0 |
| XNRR2_4438 | Branched-chain amino acid aminotransferase | 0.0 | 0.0 | | 0.0 | | 1.8 | 0.0 |
| XNRR2_4623 | 3-methyl-2-oxobutanoate hydroxymethyltransferase | 0.0 | 0.0 | | 0.0 | | 1.0 | 0.0 |
| XNRR2_4832 | 2-dehydropantoate 2-reductase | 0.0 | 0.0 | | 0.0 | | 0.0 | 0.0 |
| XNRR2_4886 | Dephospho-CoA kinase | 0.0 | 0.0 | | 0.0 | | 0.0 | 0.0 |
| XNRR2_5309 | Aminodeoxychorismate lyase | 0.0 | 0.0 | | 0.0 | | 0.0 | 0.0 |
| XNRR2_5374 | Phosphopantothenoylcysteine decarboxylase / Phosphopantothenoylcysteine synthetase | 0.0 | 0.0 | | 0.0 | | 0.0 | 0.0 |
| **Phosphotransferase system (PTS) & central carbon metabolism entry** | | | | | | | | |
| XNRR2_0026 | Transcriptional repressor of the fructose operon, DeoR family | 0.0 | 0.0 | | 0.0 | | 0.0 | 0.0 |
| XNRR2_0027 | 1-phosphofructokinase | 0.0 | 0.0 | | 0.0 | | 0.0 | 0.0 |
| XNRR2_0028 | PTS system, fructose-specific IIA component / PTS system, fructose-specific IIB component / PTS system, fructose-specific IIC component | 0.0 | -1.1 | | 0.0 | | 0.0 | 0.0 |
| XNRR2_0029 | Phosphotransferase system, phosphocarrier protein HPr | 0.0 | -1.6 | | -1.3 | | 0.0 | 0.0 |
| XNRR2_0030 | Phosphoenolpyruvate-protein phosphotransferase of PTS system | 0.0 | 0.0 | | 0.0 | | 0.0 | 0.0 |
| XNRR2_0959 | NADPH-dependent glyceraldehyde-3-phosphate dehydrogenase | 0.0 | -1.0 | | -1.6 | | 0.0 | 0.0 |
| XNRR2_0969 | Substrate binding protein | 0.0 | 0.0 | | 0.0 | | 0.0 | 0.0 |
| XNRR2_0970 | Integral membrane sugar transporter | 0.0 | -1.1 | | 0.0 | | 0.0 | 0.0 |
| XNRR2_0971 | Sugar ABC transporter permease | 0.0 | 0.0 | | 0.0 | | 0.0 | 0.0 |
| XNRR2_1023 | Phosphocarrier protein HPr | 0.0 | 0.0 | | 0.0 | | 0.0 | 0.0 |
| XNRR2_2410 | 1-phosphofructokinase | 0.0 | 0.0 | | 0.0 | | 0.0 | 0.0 |
| XNRR2_4139 | Fructose 1, 6-bisphosphatase II | 0.0 | -1.1 | | -1.9 | | 0.0 | 0.0 |
| XNRR2_4449 | Pyruvate, phosphate dikinase | 0.0 | -1.2 | | 0.0 | | 0.0 | 0.0 |
| XNRR2_4550 | Hydrolase | 0.0 | 0.0 | | -1.2 | | -1.3 | -1.6 |
| XNRR2_5287 | Phosphatase | 0.0 | 0.0 | | 0.0 | | 0.0 | 0.0 |
| XNRR2_5450 | Phosphoenolpyruvate-protein phosphotransferase of PTS system | 0.0 | 0.0 | | 0.0 | | 1.1 | 0.0 |
| XNRR2_5451 | Phosphoenolpyruvate-dependent sugar phosphotransferase | 0.0 | 0.0 | | 0.0 | | 1.3 | 0.0 |

Table S4: Gene expression of identified genes having potential regulatory functions in heterologous pamamycin producing *Streptomyces albus* J1074/R2 and *Streptomyces albus* *ΔbkdR*/R2 with supplemented l-valine in mannitol minimal medium during early growth (Early) and main production phase (Late). The expression level of the wildtype culture (J1074/R2) in minimal medium without amino acids during early growth (7h) was set as a reference. n = 3

| **Gene** | **Annotation** | **Control** | | **J1074/R2**  **+ Valine** | | ***ΔbkdR*/R2**  **+ Valine** | | |
| --- | --- | --- | --- | --- | --- | --- | --- | --- |
|  |  | **Late** | **Early** | | **Late** | | **Early** | **Late** |
| XNRR2_0615 | RNA polymerase sigma factor ECF subfamily | 0.0 | 0.0 | | 0.0 | | 0.0 | 0.0 |
| XNRR2_0683 | RNA polymerase sigma factor ECF subfamily | 0.0 | 0.0 | | 0.0 | | 0.0 | 0.0 |
| XNRR2_0749 | RNA polymerase sigma factor ECF subfamily | 0.0 | 0.0 | | 0.0 | | 0.0 | 0.0 |
| XNRR2_0776 | ROK-family transcriptional regulator | 0.0 | 0.0 | | 0.0 | | 0.0 | 0.0 |
| XNRR2_1043 | RNA polymerase, sigma 70 subunit, RpoD | 0.0 | 0.0 | | 0.0 | | 0.0 | 0.0 |
| XNRR2_1044 | Sporulation transcription factor WhiH | 4.8 | 0.0 | | 0.0 | | -3.7 | 0.0 |
| XNRR2_1071 | PpGpp synthetase/hydrolase | 0.0 | 1.2 | | 0.0 | | 0.0 | 0.0 |
| XNRR2_1132 | BldB protein | 0.0 | 0.0 | | 0.0 | | 1.0 | 0.0 |
| XNRR2_1222 | [Protein-PII] uridylyltransferase | 0.0 | -2.1 | | -2.3 | | -1.4 | -1.5 |
| XNRR2_1223 | Nitrogen regulatory protein P-II | 0.0 | -2.1 | | -2.3 | | -2.8 | -2.3 |
| XNRR2_1224 | Ammonium transporter | -1.1 | -2.3 | | -2.6 | | -2.5 | -2.8 |
| XNRR2_1225 | NsdA | 0.0 | 0.0 | | 0.0 | | 0.0 | 0.0 |
| XNRR2_1256 | Transcriptional regulator, IclR family | 0.0 | 0.0 | | 0.0 | | 0.0 | 0.0 |
| XNRR2_1391 | Neutral zinc metalloprotease | 0.0 | -2.4 | | -2.3 | | 0.0 | -1.0 |
| XNRR2_1515 | RNA polymerase ECF-subfamily sigma factor | 0.0 | 0.0 | | 0.0 | | 0.0 | 0.0 |
| XNRR2_1539 | arginine/ornithine binding protein | 0.0 | 0.0 | | 0.0 | | 0.0 | 0.0 |
| XNRR2_1554 | Nucleotide-binding protein | 5.0 | 0.0 | | 0.0 | | 0.0 | 0.0 |
| XNRR2_1574 | HTH-type transcriptional repressor dasR | 0.0 | 0.0 | | 0.0 | | 0.0 | 0.0 |
| XNRR2_1584 | RNA polymerase sigma factor RpoE, ECF subfamily | 0.0 | 0.0 | | 0.0 | | 0.0 | 0.0 |
| XNRR2_1656 | RNA polymerase sigma factor SigE, ECF subfamily | 0.0 | 1.0 | | 1.1 | | 0.0 | 0.0 |
| XNRR2_1798 | sporulation and cell division protein SsgA | 1.3 | 0.0 | | 1.1 | | 0.0 | 0.0 |
| XNRR2_1962 | Two-component system histidine kinase | 0.0 | 0.0 | | 0.0 | | 0.0 | 0.0 |
| XNRR2_1963 | Two-component system response regulator | 0.0 | 0.0 | | 0.0 | | 0.0 | 0.0 |
| XNRR2_2142 | RNA polymerase principal sigma factor hrdD | 0.0 | 0.0 | | 0.0 | | 0.0 | 0.0 |
| XNRR2_2151 | Small membrane protein | 3.5 | 0.0 | | 0.0 | | -2.3 | 0.0 |
| XNRR2_2166 | RdlB protein | 4.4 | 0.0 | | 0.0 | | 0.0 | 0.0 |
| XNRR2_2167 | RdlA protein | 5.2 | 0.0 | | 0.0 | | 0.0 | 0.0 |
| XNRR2_2231 | Transcriptional regulator AfsR | 0.0 | 0.0 | | 0.0 | | 0.0 | 0.0 |
| XNRR2_2232 | AfsS | 0.0 | 0.0 | | 0.0 | | 0.0 | 0.0 |
| XNRR2_2250 | RNA polymerase ECF-subfamily sigma factor | 0.0 | 0.0 | | 0.0 | | 0.0 | 0.0 |
| XNRR2_2306 | Factor C protein | 0.0 | 0.0 | | 0.0 | | 1.1 | 0.0 |
| XNRR2_2570 | Phosphate regulon transcriptional regulatory protein PhoB (SphR) | 0.0 | 0.0 | | 0.0 | | 0.0 | 0.0 |
| XNRR2_2571 | Phosphate regulon sensor protein PhoR (SphS) | 0.0 | 0.0 | | 0.0 | | 0.0 | 0.0 |
| XNRR2_2597 | Universal stress protein UspA | 0.0 | 0.0 | | 0.0 | | 0.0 | 0.0 |
| XNRR2_2728 | Transcriptional regulator, Crp/Fnr family | 0.0 | 0.0 | | 0.0 | | 0.0 | 0.0 |
| XNRR2_2735 | WblA | 0.0 | 0.0 | | 0.0 | | 0.0 | 0.0 |
| XNRR2_2757 | RNA polymerase ECF-subfamily sigma factor | 0.0 | 0.0 | | 0.0 | | 0.0 | 0.0 |
| XNRR2_2760 | Hypothetical protein | 0.0 | 0.0 | | 0.0 | | 0.0 | 0.0 |
| XNRR2_2769 | serine/threonine protein kinase | 0.0 | 0.0 | | 0.0 | | 0.0 | 0.0 |
| XNRR2_2903 | RNA polymerase ECF-subfamily sigma factor | 1.2 | 0.0 | | 0.0 | | -1.8 | -1.0 |
| XNRR2_2943 | SsgA | 0.0 | 0.0 | | 0.0 | | 0.0 | 0.0 |
| XNRR2_2992 | RNA polymerase sigma factor SigM, ECF subfamily | 0.0 | 0.0 | | 0.0 | | 0.0 | 0.0 |
| XNRR2_3046 | Hypothetical protein | 0.0 | 0.0 | | 0.0 | | 1.2 | 0.0 |
| XNRR2_3174 | LuxR-family transcriptional regulator | -1.6 | 0.0 | | 0.0 | | 0.0 | 0.0 |
| XNRR2_3275 | RNA polymerase ECF sigma factor | 0.0 | 0.0 | | 0.0 | | 0.0 | 0.0 |
| XNRR2_3298 | ECF subfamily RNA polymerase sigma-70 factor | 0.0 | 0.0 | | 0.0 | | 0.0 | 0.0 |
| XNRR2_3323 | GlnR-family transcriptional regulator | 0.0 | 0.0 | | 0.0 | | 0.0 | 0.0 |
| XNRR2_3489 | RNA polymerase sigma factor, ECF subfamily | 0.0 | 0.0 | | 0.0 | | 0.0 | 0.0 |
| XNRR2_3527 | BldN RNA polymerase, sigma-24 subunit, ECF subfamily | 4.3 | 0.0 | | -1.3 | | -1.7 | 0.0 |
| XNRR2_3720 | 30S ribosomal protein S12 | 0.0 | 0.0 | | 0.0 | | 0.0 | 0.0 |
| XNRR2_3805 | RpoH RNA polymerase, sigma 32 subunit, ECF subfamily | 1.4 | 0.0 | | 0.0 | | 0.0 | 0.0 |
| XNRR2_3945 | ECF sigma factor | 0.0 | 0.0 | | 0.0 | | 0.0 | 0.0 |
| XNRR2_3984 | RNA polymerase, sigma subunit, ECF family | 0.0 | 0.0 | | 0.0 | | 0.0 | 0.0 |
| XNRR2_3996 | Two-component system sensor histidine kinase AfsQ2 | 0.0 | 0.0 | | 0.0 | | 0.0 | 0.0 |
| XNRR2_3997 | Two-component system response regulator AfsQ1 | 0.0 | 0.0 | | 0.0 | | 0.0 | 0.0 |
| XNRR2_3998 | RNA polymerase ECF-subfamily sigma factor | 0.0 | 1.5 | | 1.6 | | 0.0 | 0.0 |
| XNRR2_4039 | RNA polymerase ECF-subfamily sigma factor | 0.0 | 0.0 | | 0.0 | | 0.0 | 0.0 |
| XNRR2_4181 | AraC-family transcriptional regulator | 1.2 | 0.0 | | 0.0 | | 0.0 | 0.0 |
| XNRR2_4476 | RNA polymerase, sigma 70 subunit, RpoD | 0.0 | 0.0 | | 0.0 | | 1.4 | 0.0 |
| XNRR2_4658 | Glutamine synthetase | -1.0 | -1.9 | | -2.1 | | -2.3 | -2.0 |
| XNRR2_4681 | Gamma butyrolactone receptor protein | 0.0 | 0.0 | | 0.0 | | 0.0 | 0.0 |
| XNRR2_5022 | Hypothetical protein | 0.0 | 0.0 | | 0.0 | | 0.0 | 0.0 |
| XNRR2_5117 | TetR-family transcriptional regulator | 0.0 | 0.0 | | 0.0 | | 0.0 | 0.0 |
| XNRR2_5152 | small membrane protein | 3.5 | 0.0 | | 0.0 | | 0.0 | 0.0 |
| XNRR2_5153 | secreted protein | 3.1 | 0.0 | | 0.0 | | 0.0 | 0.0 |
| XNRR2_5208 | RNA polymerase sigma factor SigK, ECF subfamily | 0.0 | 0.0 | | 0.0 | | 0.0 | 0.0 |
| XNRR2_5283 | RNA polymerase ECF-subfamily sigma factor | 0.0 | 0.0 | | 2.0 | | 1.5 | 0.0 |
| XNRR2_5315 | Sporulation and cell division protein SsgA | 0.0 | 0.0 | | 0.0 | | 0.0 | 0.0 |
| XNRR2_5340 | (P)ppGpp synthetase, SpoT/RelA | 0.0 | 0.0 | | 0.0 | | 0.0 | 0.0 |
| XNRR2_5362 | Pleiotropic negative regulator BldD | 0.0 | 0.0 | | 0.0 | | 0.0 | 0.0 |
| XNRR2_5529 | RNA polymerase ECF-subfamily sigma factor | 0.0 | 0.0 | | 0.0 | | 0.0 | 0.0 |
| XNRR2_5625 | RNA polymerase, sigma-24 subunit, ECF subfamily | 0.0 | 3.5 | | 3.1 | | 2.4 | 0.0 |
| XNRR2_5652 | RNA polymerase sigma factor SigL, ECF subfamily | 0.0 | 0.0 | | 1.9 | | 1.6 | 0.0 |
| XNRR2_5893 | ECF subfamily RNA polymerase sigma factor | 0.0 | -2.6 | | -2.5 | | -2.3 | 0.0 |

**Table S5:** Gene expression of phosphopantetheinyl transferases in heterologous pamamycin producing *Streptomyces albus* J1074/R2 and *Streptomyces albus* *ΔbkdR*/R2 with supplemented L-valine in mannitol minimal medium during early growth (Early) and main production phase (Late). The expression level of the wildtype culture (J1074/R2) in minimal medium without amino acids during early growth (7h) was set as a reference. n = 3

| **Gene** | **Annotation** | **Control** | | **J1074/R2**  **+ Valine** | | ***ΔbkdR*/R2**  **+ Valine** | | |
| --- | --- | --- | --- | --- | --- | --- | --- | --- |
|  |  | **Late** | **Early** | | **Late** | | **Early** | **Late** |
| XNRR2_0579 | 4'-phosphopantetheinyl transferase | 0.0 | 0.0 | | 0.0 | | 0.0 | 0.0 |
| XNRR2_1238 | Phosphopantetheine adenylyltransferase | 0.0 | 0.0 | | 0.0 | | 0.0 | 0.0 |
| XNRR2_5716 | Sfp-type phosphopantetheinyl transferase | 0.0 | 0.0 | | 0.0 | | 0.0 | 0.0 |

**Table S6:** Gene expression of acetyl-CoA carboxylases and transcriptional regulator *AccR* homolog in heterologous pamamycin producing *Streptomyces albus* J1074/R2 and *Streptomyces albus* *ΔbkdR*/R2 with supplemented L-valine in mannitol minimal medium during early growth (Early) and main production phase (Late). The expression level of the wildtype culture (J1074/R2) in minimal medium without amino acids during early growth (7h) was set as a reference. n = 3

| **Gene** | **Annotation** | **Control** | | **J1074/R2**  **+ Valine** | | ***ΔbkdR*/R2**  **+ Valine** | | |
| --- | --- | --- | --- | --- | --- | --- | --- | --- |
|  |  | **Late** | **Early** | | **Late** | | **Early** | **Late** |
| XNRR2_2273 | Acetyl/propionyl CoA carboxylase alpha subunit | 0.0 | 0.0 | | 0.0 | | 0.0 | 0.0 |
| XNRR2_2274 | Acetyl/propionyl CoA carboxylase, beta subunit | 0.0 | 0.0 | | 0.0 | | 0.0 | 0.0 |
| XNRR2_4211 | Acetyl/propionyl CoA carboxylase alpha subunit | 0.0 | 2.8 | | 3.1 | | 4.6 | 3.4 |
| XNRR2_4212 | Acetyl/propionyl CoA carboxylase | 0.0 | 2.6 | | 2.7 | | 4.3 | 2.9 |
| XNRR2_4213 | TetR-family transcriptional regulator | 0.0 | 1.6 | | 1.1 | | 0.0 | 0.0 |


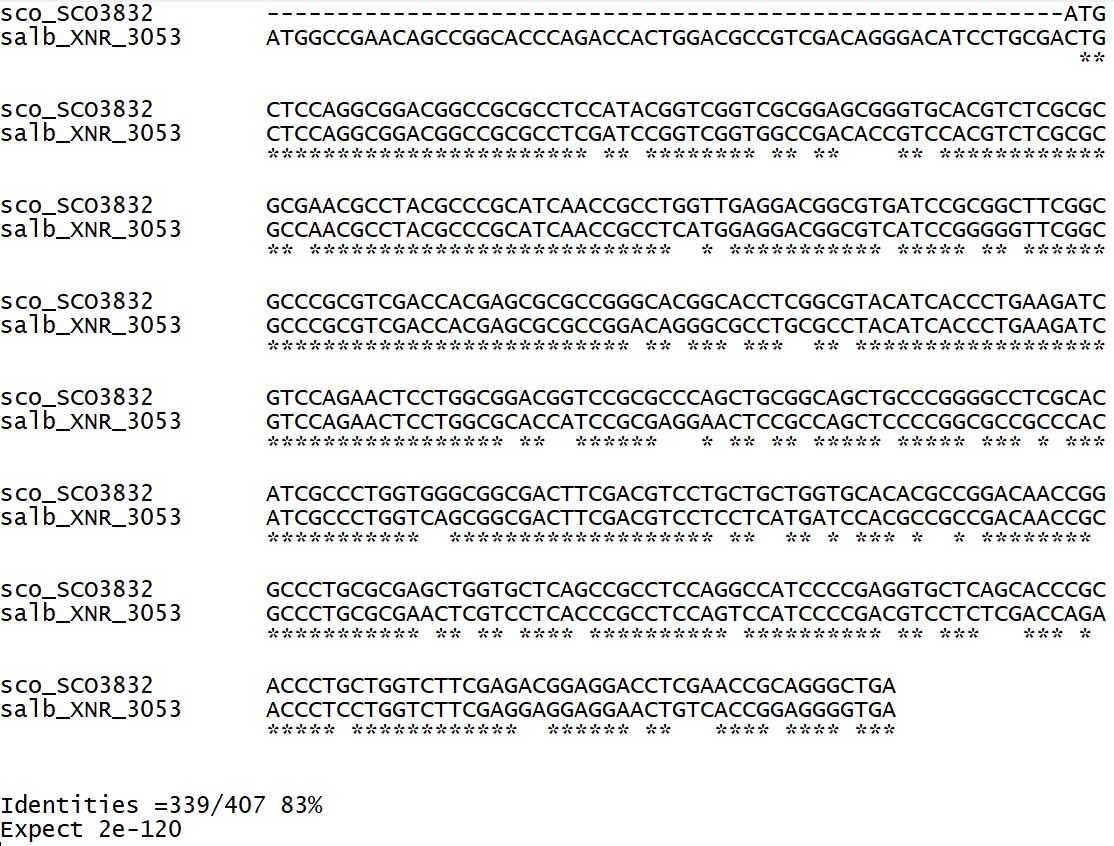


**Figure S1:** Alignment of the gene encoding the branched-chain amino acid dehydrogenase regulator *bkdR* of *Streptomyces coelicolor* (sco_SCO3832) and its homolog salb_XNR_3053 in *Streptomyces albus* J1074. The sequences exhibit a homology of 83% and an expected value of 2e-120.


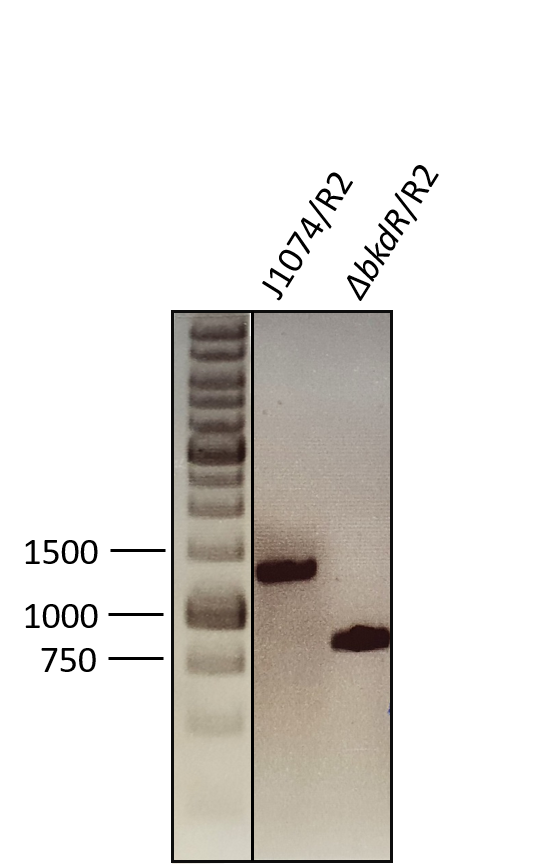


**Figure S2:** Verification of the deletion of XNRR2_3053 in *Streptomyces albus* J1074/R2 using PCR. The primer pair 3053_ch_Fw and 3053_ch_rev was used to prove the deletion.


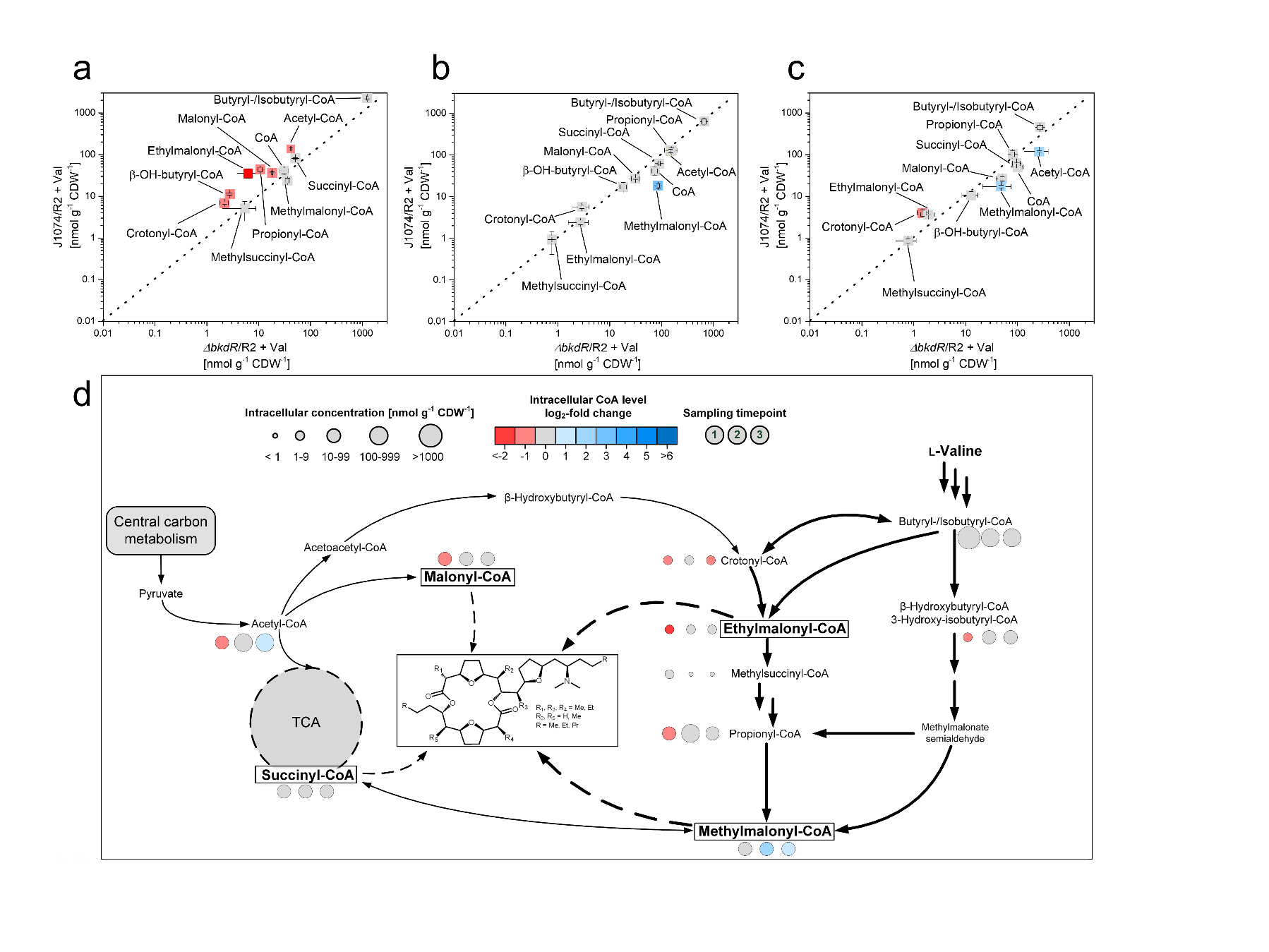


**Figure S3**: **Dynamics of intracellular CoA thioesters during pamamycin production in *Streptomyces albus* J1074/R2 *ΔbkdR***. The data show the correlation of absolute levels between wildtype (J1074/R2) and mutant at three different timepoints (a, b, and c). In addition, the data are mapped on the pathways of CoA thioester metabolism, whereby the size of the given circles represents the absolute concentrations at each timepoint (1, 2, 3), and the color represents the respective log_2_-fold change, as compared to the wild type /R2. n = 3





**Figure S4:** Molecular weight distribution of pamamycin, newly produced during the stationary phase by *Streptomyces albus* J1074/R2 *ΔbkdR* on a mannitol-based medium, supplemented with 3 mM l-valine. The spectrum is inferred by differential calculation, considering the pamamycin spectrum at the end of the growth phase (28 h) when all carbon was depleted and at the process end after 48 hours. n = 3


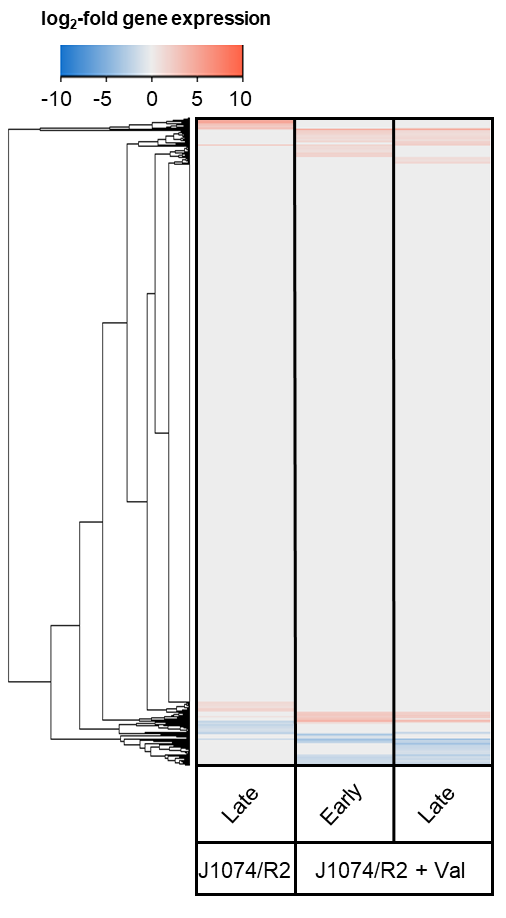


**Figure S5:** Hierarchical cluster analysis of global gene expression of *S. albus* J1074/R2 on a minimal mannitol medium, supplemented with 3 mM l-valine and without l-valine. Samples were taken after 7 h (Early) and 18 h (Late). The gene expression of the control culture during early growth (7 h) was set as reference. n = 3


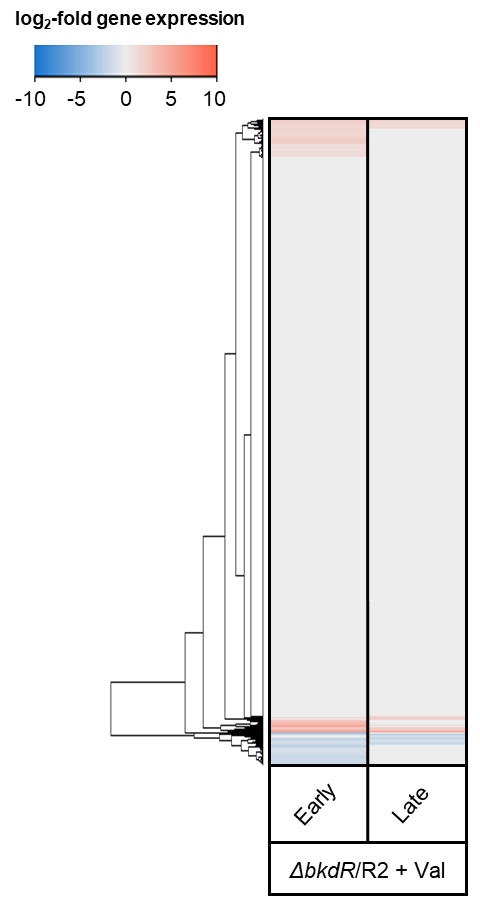


**Figure S6:** Hierarchical cluster analysis of global gene expression of *S. albus* J1074/R2 *ΔbkdR* on a minimal mannitol medium, supplemented with 3 mM l-valine. Samples were taken after 7 h (Early) and 18 h (Late). The gene expression of the control (wild type without l-valine) during early growth (7 h) was set as reference. n = 3

**
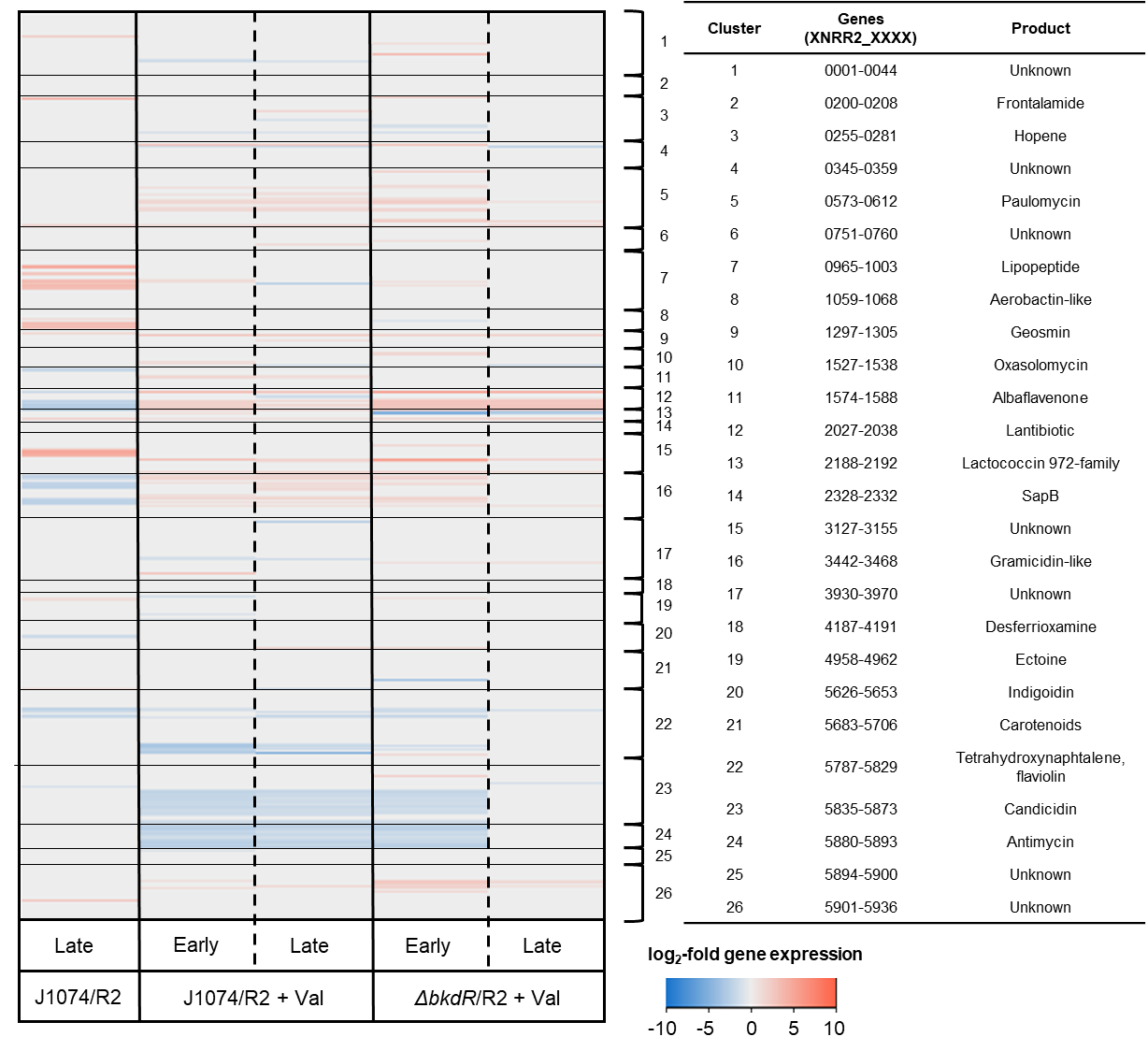
**

**Figure S7:** Gene expression of secondary metabolite clusters of *S. albus* J1074/R2 and its mutant *S. albus* J1074/R2 *ΔbkdR* in minimal mannitol medium, partially supplemented with 3 mM l-valine (+Val). Samples were taken after 7 h (Early) and 18 h (Late). The identified secondary metabolite clusters relate to previous work [[3](#_ENREF_3)]. The gene expression of the control (wild type without l-valine) during early growth (7 h) was set as reference. n = 3

**
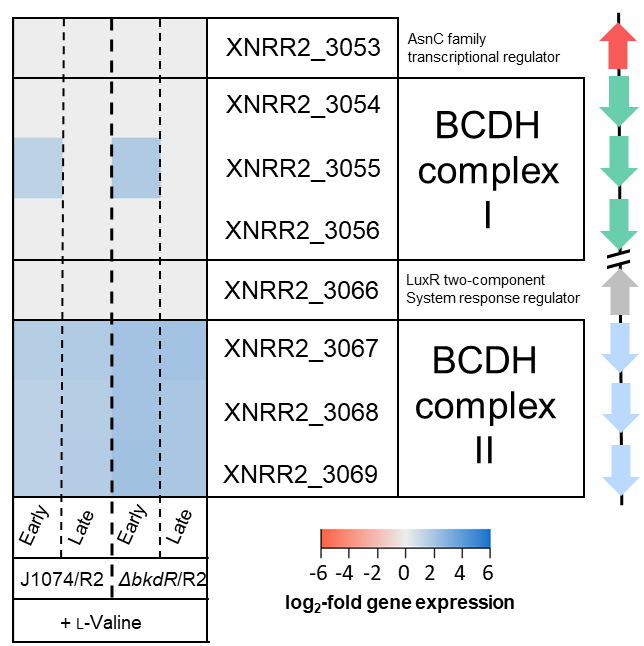
**

**Figure S8:** Gene expression of the two branched-chain amino acid dehydrogenase (BCDH) complexes in *Streptomyces albus* J1074/R2 and *S. albus* J1074/R2 *ΔbkdR* in minimal mannitol medium, supplemented with 3 mM l-valine. Samples were taken after 7 h (Early) and 18 h (Late). The gene expression of the control (wild type without l-valine) during early growth (7 h) was set as reference. n = 3


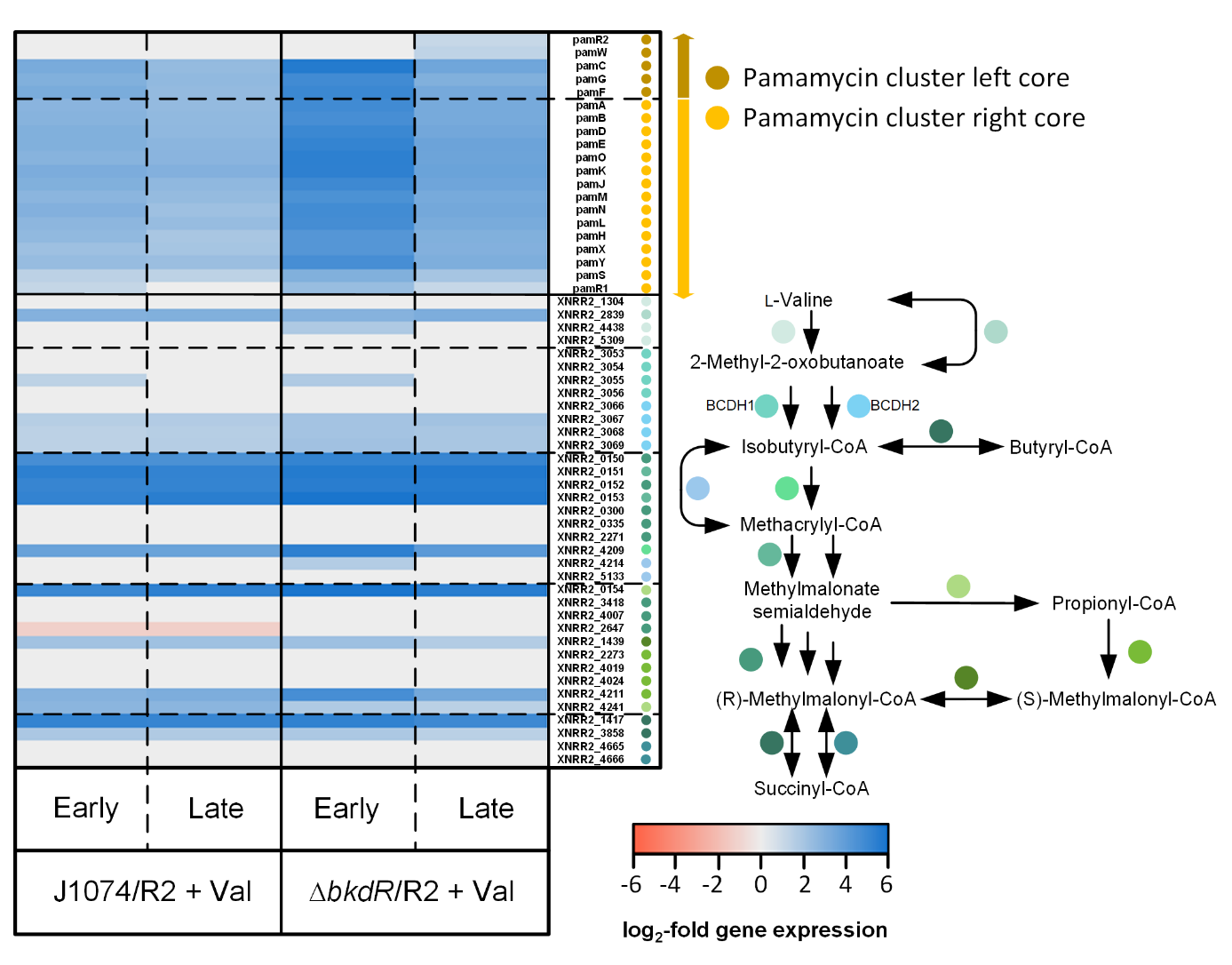


**Figure S9:** Expression of the pamamycins biosynthetic gene cluster and genes related to l-valine degradation in *Streptomyces albus* J1074/*R2* and *Streptomyces albus* *ΔbkdR*/R2. The pamamycin cluster genes are sorted according to their affiliation of the individual cores (left and right core) [[4](#_ENREF_4)]. The l-valine degradation genes are sorted in relation to the KEGG-based pathway. Samples were taken after 7 h (Early) and 18 h (Late). The gene expression of the control (wild type without l-valine) during early growth (7 h) was set as reference. n = 3


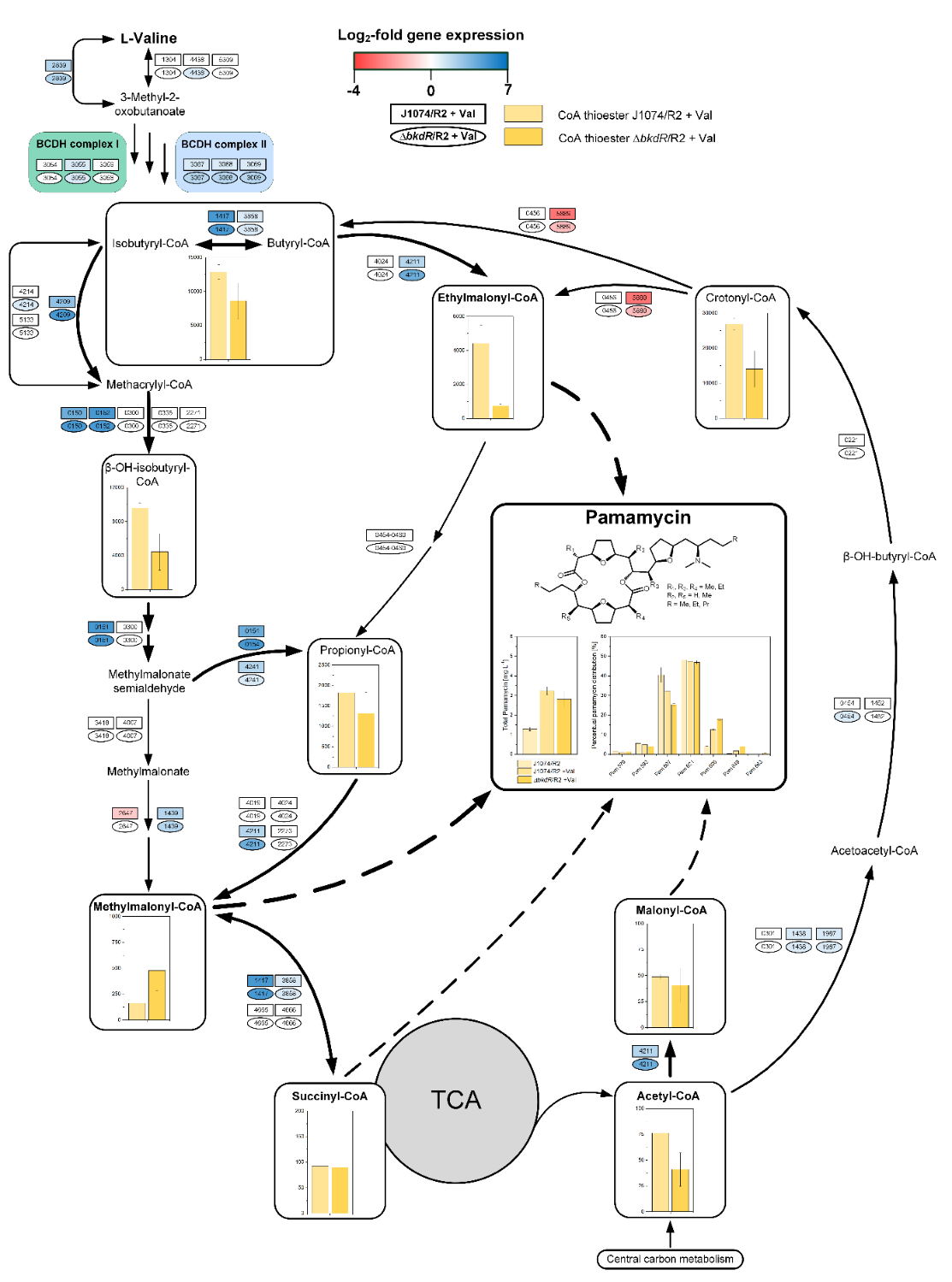


**Figure S10:** Multi-omics view on the effect of supplemented l-valine on pamamycin biosynthesis and supporting pathways in *Streptomyces albus* J1074/R2 and its mutant *Streptomyces albus* *ΔbkdR*/R2. The box colors display the differential gene expression of J10747R2 (rectangle) and its mutant (ellipse) during the early growth phase (7 h). The numbers inside the boxes indicate the specific gene number (XNRR2_XXXX). The bar charts display the relative intracellular CoA thioester levels at the same time point. The values from the wild type without l-valine (Control) was set to 100%.


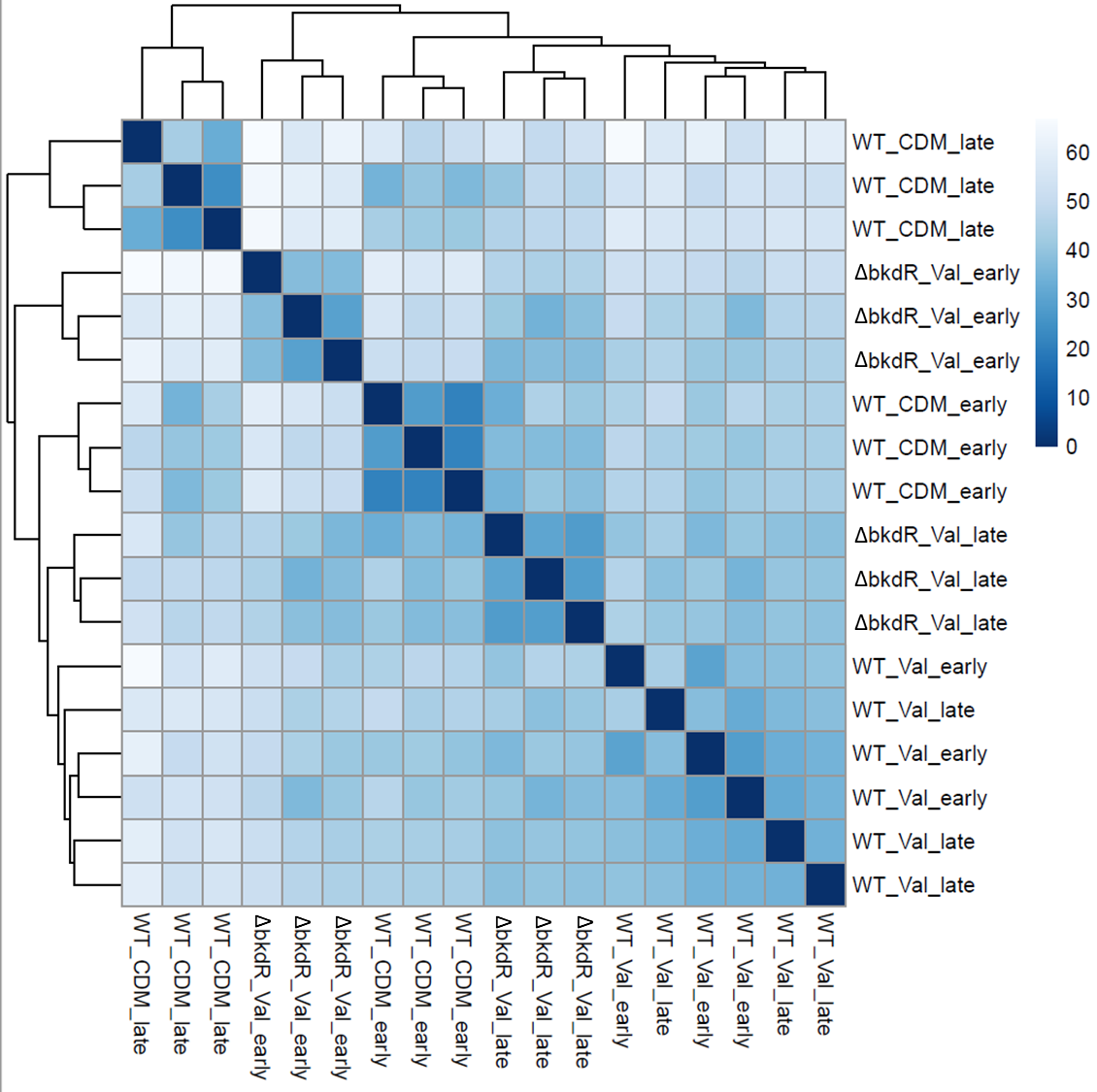


**Figure S11:** Heatmap of the sample-to-sample distances from the RNA sequencing data. The background corrected read count data were fed to DESeq2 [[5](#_ENREF_5)] to calculate normalized read counts. After regularized log transformation with blind dispersion estimation enabled. the sample-to-sample distances were calculated and used for hierarchical clustering. which in turn was visualized using pheatmap [[6](#_ENREF_6)].


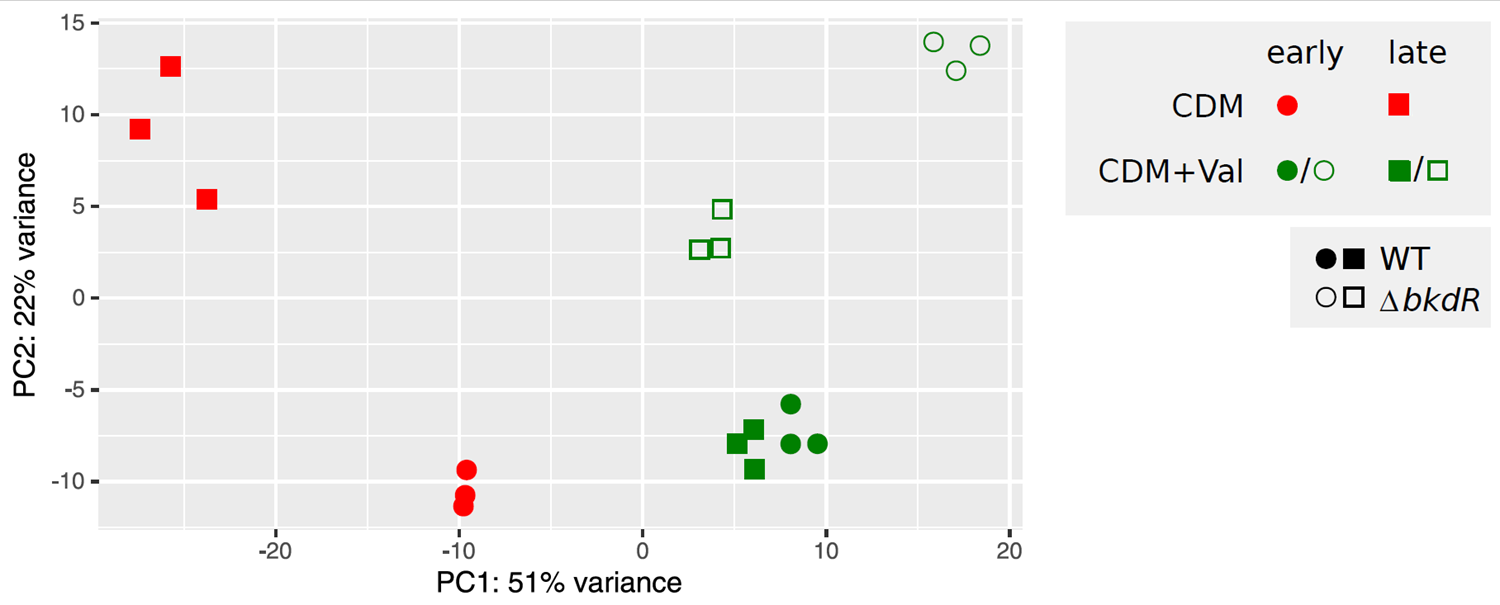


**Figure S12:** PCA of the RNA seq data**.** The background corrected read count data were fed to DESeq2 to calculate normalized read counts. After regularized log transformation with blind dispersion estimation enabled. a PCA was performed and visualized using ggplot2 [[7](#_ENREF_7)].

**Literature**

1. Kieser T. Bibb M. Buttner M. Chater K. Hopwood D: **Practical Streptomyces Genetics** *John Innes Foundation. Norwich. United Kingdom* 2000.

2. Barton N. Horbal L. Starck S. Kohlstedt M. Luzhetskyy A. Wittmann C: **Enabling the valorization of guaiacol-based lignin: Integrated chemical and biochemical production of cis.cis-muconic acid using metabolically engineered *Amycolatopsis* sp ATCC 39116.** *Metabolic engineering* 2018. **45:**200-210.

3. Lopatniuk M. Myronovskyi M. Nottebrock A. Busche T. Kalinowski J. Ostash B. Fedorenko V. Luzhetskyy A: **Effect of "ribosome engineering" on the transcription level and production of *S. albus* indigenous secondary metabolites.** *Applied microbiology and biotechnology* 2019. **103:**7097-7110.

4. Kuhl M. Gläser L. Rebets Y. Rückert C. Sarkar N. Hartsch T. Kalinowksi J. Luzhetskyy A. Wittmann C: **Microparticles globally reprogram the metabolism of *Streptomyces albus* towards accelerated morphogenesis. streamlined carbon core metabolism and enhanced production of the antituberculosis polyketide pamamycin.** *Biotechnology & Bioengineering* 2020. ***submitted***.

5. Love MI. Huber W. Anders S: **Moderated estimation of fold change and dispersion for RNA-seq data with DESeq2.** *Genome biology* 2014. **15:**550.

6. Kolde R: **Pheatmap: Pretty Heatmaps. R Package Version 1.0.12.** 2019.

7. Wickham H: **ggplot2: elegant graphics for data analysis.** *Springer* 2016.
